# Gene flow as a simple cause for an excess of high‐frequency‐derived alleles

**DOI:** 10.1111/eva.12998

**Published:** 2020-06-02

**Authors:** Nina Marchi, Laurent Excoffier

**Affiliations:** ^1^ CMPG Institute of Ecology and Evolution University of Berne Berne Switzerland; ^2^ Swiss Institute of Bioinformatics Lausanne Switzerland

**Keywords:** computer simulation, demographic analysis, gene flow, human genetics, human genome, natural selection, neutral evolution

## Abstract

Most human populations exhibit an excess of high‐frequency variants, leading to a U‐shaped site‐frequency spectrum (uSFS). This pattern has been generally interpreted as a signature of ongoing episodes of positive selection, or as evidence for a mis‐assignment of ancestral/derived allelic states, but uSFS has also been observed in populations receiving gene flow from a ghost population, in structured populations, or after range expansions. In order to better explain the prevalence of high‐frequency variants in humans and other populations, we describe here which patterns of gene flow and population demography can lead to uSFS by using extensive coalescent simulations. We find that uSFS can often be observed in a population if gene flow brings a few ancestral alleles from a well‐differentiated population. Gene flow can either consist in single pulses of admixture or continuous immigration, but different demographic conditions are necessary to observe uSFS in these two scenarios. Indeed, an extremely low and recent gene flow is required in the case of single admixture events, while with continuous immigration, uSFS occurs only if gene flow started recently at a high rate or if it lasted for a long time at a low rate. Overall, we find that a neutral uSFS occurs under more restrictive conditions in populations having received single pulses of gene flow than in populations exposed to continuous gene flow. We also show that the uSFS observed in human populations from the 1000 Genomes Project can easily be explained by gene flow from surrounding populations without requiring past episodes of positive selection. These results imply that uSFS should be common in non‐isolated populations, such as most wild or domesticated plants and animals.

## 1 INTRODUCTION

Allele frequency changes are driven by the combined action of different evolutionary forces such as mutation, selection, genetic drift and migration (Wright, 1931), but most of the variant frequencies are expected to be rare (Ewens, 1972), leading to a L‐shaped site‐frequency spectrum (SFS) (Fu, 1995). However, an unexpectedly large proportion of high‐frequency‐derived alleles, resulting in U‐shaped SFS (uSFS), has been documented in multiple species, including wild and domesticated plants (Liu, Zhou, Morrell, Gaut, & Ge, 2017; Morton, Dar, & Wright, 2009; Price et al., 2018), animals (Cooper, Burrus, Ji, Hahn, & Montooth, 2015; de Manuel et al., 2016; Murray, Huerta‐Sanchez, Casey, & Bradley, 2010) and even human populations (Henn et al., 2015; Pouyet, Aeschbacher, Thiéry, & Excoffier, 2018).

Several explanations for these uSFS have been proposed. This phenomenon has been notably interpreted as a signature of positive selection at several loci (Akashi & Schaeffer, 1997; Bustamante, Wakeley, Sawyer, & Hartl, 2001), as neutral variants hitchhiking with beneficial mutations during selective sweeps would also be observed at high frequencies (Andolfatto & Przeworski, 2001; Fay & Wu, 2000; Kim & Stephan, 2000, 2002; Lapierre, Blin, Lambert, Achaz, & Rocha, 2016; Pavlidis, Jensen, & Stephan, 2010; Stephan, 2016), thus leading to a uSFS (Hahn, 2018; Ronen, Udpa, Halperin, & Bafna, 2013). This phenomenon could even be accentuated by selection fluctuating over time (Huerta‐Sanchez, Durrett, & Bustamante, 2008; Przeworski, 2002). Alternatively, low‐frequency‐derived alleles mistakenly annotated as ancestral would lead to the emergence of high‐frequency‐derived variants and also create a uSFS (Baudry & Depaulis, 2003; Hernandez, Williamson, & Bustamante, 2007). uSFS can also emerge in multiple‐merger coalescent models that have been developed to account for strong selective sweeps or a very large variance in reproductive success among individuals of a population (Eldon, Birkner, Blath, & Freund, 2015; Rice, Novembre, & Desai, 2018; Sargsyan & Wakeley, 2008; Tellier & Lemaire, 2014), which is not well accounted for in the classical Kingman coalescent framework. Finally, uSFS has also been shown to arise in non‐isolated populations, for example in range expanding populations (Sousa, Peischl, & Excoffier, 2014), in structured populations analysed as single populations (Cutter, 2019; Lapierre et al., 2016; Wakeley, 2000) or in structured population receiving low levels of gene flow from surrounding demes (Garrigan & Hammer, 2006; Wakeley & Aliacar, 2001).

Even though most animal and plant populations are not completely isolated and receive migrants from surrounding populations, gene flow has been rarely proposed as an explanation for uSFS, and hypotheses of selection or ancestral allele mis‐assignment have been preferred (Li et al., 2012; Liu et al., 2017; Qanbari & Simianer, 2014; Sabeti, 2006). However, Pouyet et al. (2018) recently showed that the uSFS observed in human populations could not be recovered under a complex demographic scenario involving an isolated population, but could be perfectly modelled under a scenario involving gene flow from an unspecified source (i.e. a ghost population), and this, in absence of any positive selection or mis‐assignment of ancestral alleles. In order to better investigate the conditions leading to uSFS in non‐isolated populations, we have used simulations to explore the impact of gene flow duration, onset and intensity, as well as of population size and divergence time, on the probability of observing a uSFS. Even though more complex scenarios could certainly lead to uSFS, we have simulated here two simple demographic models of *isolation with admixture* and of *isolation with immigration*, which are often used as basic population genetic models (Geneva & Garrigan, 2010; Hahn, 2018; Patterson et al., 2012; Sousa & Hey, 2013) and represent the two ends of the gene flow spectrum. We have then compared the likelihoods of these models for ten populations from the 1,000 Genomes panel where uSFS is observed.

## 2 MATERIAL AND METHODS

### 2.1 Simulated scenarios

**FIGURE 1:**
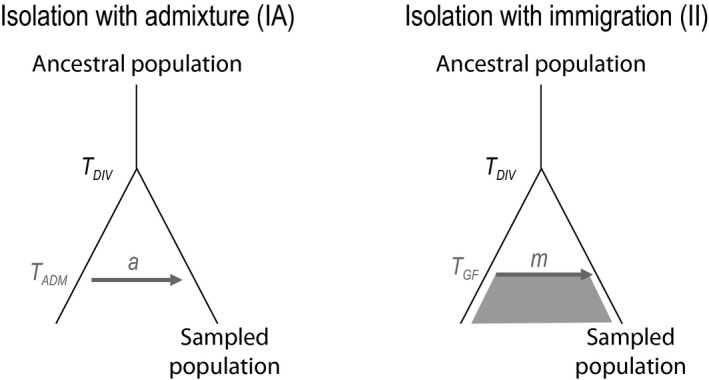
Scenarios used to elucidate conditions under which gene flow leads to a uSFS. The populations have diverged *T*_DIV_ generations ago from an ancestral population; their population sizes (*N*) are identical and constant over time. The results shown in the main text were obtained for a sampling size of 10 individuals and population sizes *N* = 4,000, but similar results were seen for a ten‐time larger size (*N* = 40,000) after appropriate rescaling of divergence time and migration rate (Supporting Information 1)

We modelled a population of *n* haploid individuals and effective size *N* that receives gene flow from an unsampled and often referred to as a “ghost” population (Beerli, 2004; Slatkin, 2005), after their divergence *T*_DIV_ generations ago (or expressed in 2*N* units as $\tau_{\text{DIV}}=T_{\text{DIV}}/\left(2N\right)$ the parameters and their ranges are described in Table 1). However, rather than being simply a non‐sampled population, this ghost population is introduced here as a convenient way to partition sampled lineages into two structured components between which coalescent events will not immediately occur. This type of partitioning is for instance found in metapopulation models with migration, where coalescent events occur rapidly during a scattering phase and more slowly during the collecting phase (Wakeley, 1999; Wakeley & Aliacar, 2001). For sake of simplicity, we tested two models at the ends of the gene flow spectrum (Figure 1): one of *isolation with admixture* (IA) and one of *isolation with immigration* (*II*). In the *IA* model, a single admixture event (i.e. a single pulse of gene flow) occurred *T*_ADM_ generations ago ( $\tau_{\text{ADM}}$ in 2*N* units), with an admixture rate *a*. In the *II* model, continuous gene flow occurring at rate *m* per generation started *T*_GF_ generations ago.

**TABLE 1:** Parameters description and ranges used for the simulation of different scenarios

Parameter description	*IA* model	*II* model
Effective size (haploid number, *Ns*)	{4,000; 40,000}	{4,000; 40,000}
Divergence time $\tau_{\text{DIV}}=T_{\text{DIV}}/\left(2N\right)$	{0.005; 0.05; 0.25; 0.5; 2.5}	2.5
Time of single admixture event $\tau_{\text{ADM}}=T_{\text{ADM}}/\left(2N\right)$	{0; 0.025; 0.05; 0.125; 0.25}	
Onset of gene flow (*T*_GF_)		*T*_DIV_/ {10,000; 1,000; 100; 10; 1}
Haploid sample size (*n*)	{10; 50}	10
Admixture rate (*a*)	[0:0.5]	
Immigration rate (*Nm*)		[0.01:10]

### 2.2 Simulated genetic data

We used the software *fastsimcoal2* (Excoffier, Dupanloup, Huerta‐Sánchez, Sousa, & Foll, 2013) to simulate the genomic diversity in 100 Mb of DNA (which roughly corresponds to the neutral portion of the human genome found in Pouyet et al. (2018)) under the scenarios defined in the previous section. The simulated 100 Mb was modelled as 10,000 blocks of 1,000 independent non‐recombining regions of 100 bp. Note that the SNPs simulated in this way are essentially independent (unlinked) SNPs, and that it would have been possible to simulate partially linked SNPs but more simulations would have been necessary to get the same expected SFS (Pouyet et al., 2018). However, we have performed a limited set of simulation using partially linked SNPs, to verify that our conclusions would not change if we were explicitly simulating linkage and recombination (Supporting Information 2).

We then computed the site‐frequency spectrum (SFS) for each block independently using the *fastsimcoal2* command./fsc2 –i *File.par* ‐n 10,000 ‐q ‐c0 ‐d ‐s0 ‐x ‐I (Supporting Information 3). The mutation rate was set to 1.20 × 10^–8^ per bp per generation (de Manuel et al., 2016; Venn et al., 2014), and we assumed an infinite‐site model. We then sampled with replacement 10,000 blocks from the original simulated set to generate a given block‐bootstrap data set, and we repeated this procedure 1,000 times to generate 1,000 block‐bootstrap SFS.

### 2.3 Summary statistics

We computed the global unfolded SFS for each simulated and block‐bootstrapped data set of 100 Mb, by summing the 10,000 (respectively, observed or randomly sampled) block‐SFS. The 95% confidence intervals of the simulated SFS were computed from the 2.5% and 97.5% quantiles of the SFS entries (all SFS is shown in Supporting Information 4).

**FIGURE 2:**
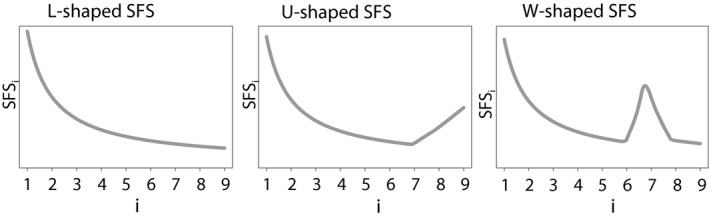
Schematic SFS shapes, for a sample of haploid size 10 where ${\text{SFS}}_{i}$ is the number of sites with a derived frequency *i*

We classified simulated SFS into three categories according to their shapes: a monotonously decreasing SFS with a mode at singletons corresponding to a L‐shape SFS; a U‐shape SFS with a second mode at high derived allele frequencies; a W‐shape SFS with a second mode at intermediate derived allele frequencies (Figure 2).

We also used a summary statistic called *D‐tail* defined as.

$D-tail=\frac{{\text{SFS}}_{n-1}-{\text{SFS}}_{n-2}}{{\text{SFS}}_{n-2}}$ , where *n* is the haploid sample size and ${\text{SFS}}_{i}$ is the number of sites with a derived frequency *i*. *D‐tail* is positive when ${\text{SFS}}_{n-1}$ > ${\text{SFS}}_{n-2}$ (i.e. for uSFS) and negative for L‐shaped and W‐shaped SFS.

### 2.4 Human data sets and likelihood estimations

We computed the SFS and *D‐tail* statistic for ten 1,000 Genomes (1000G) populations: Yoruba in Ibadan, Nigeria (YRI); Luhya in Webuye, Kenya (LWK); Iberian Population in Spain (IBS); British in England and Scotland (GBR); Punjabi from Lahore, Pakistan (PJL); Bengali from Bangladesh (BEB); Kinh in Ho Chi Minh City, Vietnam (KHV); Japanese in Tokyo, Japan (JPT); Colombians from Medellin, Colombia (CLM); and Peruvians from Lima, Peru (PEL). We included the 10 individuals with the highest coverage per population (see Supporting table from Pouyet et al. (2018)). In this data set, we focused on the 493,369 sites formerly identified as evolving neutrally in Pouyet et al. (2018) (i.e. biallelic sites, non‐CpG sites, mutations neither affected by biased gene conversion nor by background selection). The ancestral state was defined based on the chimpanzee reference genome (*panTro4*) to prevent mis‐assignment of the ancestral/derived states. We used a block‐bootstrap approach based on sets of 100 adjacent SNPs along the genome, to generate 1,000 block‐bootstrap SFS and *D‐tail* statistics.

We estimated with *fastsimcoal2* the likelihood of four demographic scenarios (Supporting Information 5) inspired from Pouyet et al. (2018): (a) a first simple scenario, where a fully isolated population can go through four different epochs with four different sizes separated by three bottlenecks of arbitrary size and times, (b) same as the first scenario but allowing for potential ancestral‐state mis‐assignment (option ‐ASM in *fastsimcoal*), (c) same as the first scenario but allowing for continuous gene flow from a ghost population and (d) same as the first scenario but allowing for a single pulse of admixture from a ghost population. Parameters were estimated for each model with the *fastsimcoal2* command line options: ‐t *POP*.tpl ‐e *POP*.est ‐n200000 ‐d ‐M ‐L40 ‐q ‐0 ‐C1 ‐c1 ‐B1, where *POP* is the acronym of each of the ten 1000G populations (generic input files made available in Supporting Information 6). In order to scale parameters, we assumed an ancestral human population size of 20,000 diploids, and a constant and uniform mutation rate of 1.25 × 10^–8^ per bp per generation (Scally & Durbin, 2012), which is widely used in demographic inference in humans (Malaspinas et al., 2016; Pagani et al., 2015; Raghavan et al., 2015; Schiffels & Durbin, 2014; Sikora et al., 2017, 2019; Spence & Song, 2019; Steinrücken, Kamm, Spence, & Song, 2019).

## 3 RESULTS

### 3.1 Isolation with admixture

To evaluate the impact of the divergence time, we have first simulated an *isolation with admixture* (IA) model where the admixture event occurred at sampling time (0 generations ago) for varying divergence times (*T*_DIV_) and admixture rates (*a*).

**FIGURE 3:**
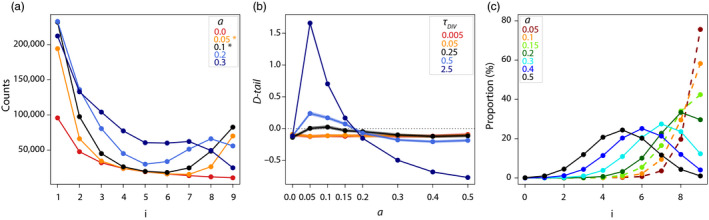
Effect of the admixture rate and time of divergence on SFS properties, under an *IA* scenario for $\tau_{\text{ADM}}=0$ . (a) SFS, i.e. the number of sites with a derived frequency *i,* from *n *= 10 haploid individuals for $\tau_{\text{DIV}}=2.5$ and various admixture rates *a*; (b) *D‐tail* statistic for various divergence times $\tau_{\text{DIV}}$ ; (c) proportion of loci in the sampled population that were fixed for the derived allele before the admixture event and which show *i* derived alleles afterwards, when $\tau_{\text{DIV}}=2.5$ . In panes a and b, dots and solid lines were obtained from simulated data sets, and semi‐transparent colours define 95% block‐bootstrap confidence intervals. Note that these confidence intervals are so small that they are barely visible on these figures. In pane c, dashed lines stand for uSFS and solid lines stand for W‐shaped SFS

As expected, without admixture (*a* = 0), the SFS is L‐shaped (Figure 3a and Supporting Information 7) and the D‐tail statistics are negative (Figure 3b). This is also the case when *a >* 0 for recent divergence times ( $\tau_{\text{DIV}}=T_{\text{DIV}}/\left(2N\right)$ = 0.005 or $\tau_{\text{DIV}}$ = 0.05). However, for older divergence times, when $\tau_{\text{DIV}}>0.05$ , the pattern is more complex: positive *D‐tail* statistics and consistent uSFS are only observed for relatively low admixture rates (between 5% and 20%). Importantly, the admixture rates leading to uSFS depend on the sample size *n*: for a larger sample size (*n* = 50), we observe uSFS for reduced admixture rates (0 < *a* ≤ 0.03), while larger admixture rates lead to W‐shaped SFS with not only one but two internal maxima (Supporting Information 8). In any case, independently of sample sizes, *D‐tail* values increase for older divergence times, indicating that SFS is more strongly U‐shaped with larger divergence times.

These results are best explained by the immigration of a few ancestral alleles into the sampled population at sites where the derived allele is fixed in the sample before admixture, thus causing a decrease in the frequency of derived alleles from *n* to *n*‐1. For the same amount of admixture, this phenomenon is more likely if two populations have fixed different alleles, the probability of which increases with divergence times, and becomes substantial when $\tau_{\text{DIV}}\geq 0.5$ (Hudson & Coyne, 2002). To substantiate this explanation, we have performed simulations for $\tau_{\text{DIV}}=2.5$ , where we computed derived allele frequencies after the admixture event at sites that were fixed‐derived before admixture (Figure 3c). For relatively low admixture rates (*a *= 0.05), almost 80% of the previously fixed derived sites are transformed into nearly fixed sites and SFS becomes U‐shaped. This proportion drops to 60% when *a *= 0.1. For larger admixture rates (*a *≥ 0.2), SFS becomes W‐shaped (Supporting Information 7), as admixture events will often introduce more than one ancestral allele at previously fixed sites.

Note that under the *IA* model, large admixture rates corresponding to partial genetic replacement (0.5 < *a* < 1) can also lead to uSFS. Indeed, uSFS is also obtained for admixture rates between 0.8 and 0.95, in a way symmetrical to low *a* values (0.05 < *a* < 0.2, Supporting Information 9A). In this case, the excess of high derived frequencies is caused by the immigration of a large number of derived alleles at sites where the ancestral allele was fixed in the sampled population (Supporting Information 9B), mimicking the action of positive selection (Hahn, 2018).

### 3.2 Effects of the onset time of instantaneous and continuous gene flow

When gene flow occurred more than one generation ago, its onset time, intensity and duration might also have a drastic effect on the allele frequency distribution, and thus on the shape of the SFS. To investigate the effect of past gene flow, we have run simulations under both an *isolation with admixture* (IA) and an isolation with immigration (*II*) models, where the populations have diverged for $\tau_{\text{DIV}}=2.5$ (i.e. 20,000 generations for effective populations size *N* = 4,000), when $\tau_{\text{ADM}}>0$ and when *T*_GF_ > 0 for the* IA* and *II* models, respectively.

**FIGURE 4:**
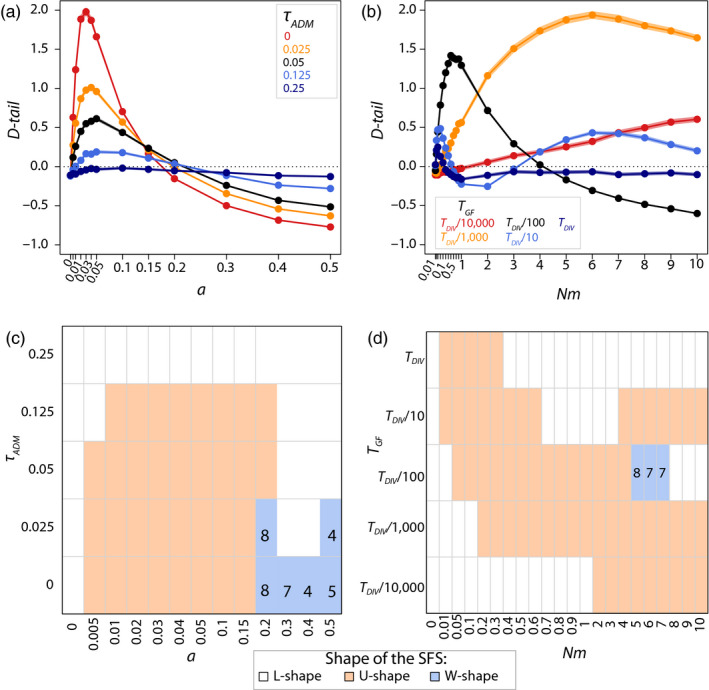
Effect of rate and age of gene flow on SFS properties, for $\tau_{\text{DIV}}=2.5$ and *n = *10 under an *IA* model with different admixture times ( $\tau_{\text{ADM}}$ ) and rates (*a*) (left panes) or under an *II* model with different gene flow onset (*T*_GF_) and number of migrants per generation (*Nm*) (right panes). *D‐tail* statistic (panes a and b) with dots and solid lines obtained from simulated data sets, and semi‐transparent colours defining the 95% confidence intervals calculated from the block‐bootstrap data sets (note that these confidence intervals are so small that they are barely visible on these figures); SFS shapes (panes c and d) with black numbers indicating the derived frequency *i* of the internal mode of W‐shaped SFS

Under the *IA* model, uSFS and positive *D‐tail* values are observed for admixture $\tau_{\text{ADM}}<0.25$ . SFS becomes less U‐shaped, and *D‐tail* values are smaller for older admixture times (Figure 4a). However, even though *D‐tail* statistics are lower when admixture is old (i.e. $\tau_{\text{ADM}}=0.125$ ), uSFS is observed for larger admixture rates than when it is very recent (i.e. when $\tau_{\text{ADM}}=0$ ). As expected, the SFS can become multimodal for recent divergence times and large admixture rates ( $\tau_{\text{ADM}}\leq 0.025$ and *a* ≥ 0.2), and the internal mode moves towards more central values for larger admixture rates (Figure 4c).

Under the *II* model, we observe uSFS and positive *D‐tail* statistics for a large range of onset times for gene flow (from *T*_GF_ = *T*_DIV_ to *T*_GF_ = *T*_DIV_/10,000; Figure 4b and d), but the amount of gene flow required to produce uSFS is inversely correlated with the age of the onset of gene flow; that *is,* small immigration rates (*Nm* < 0.5) are necessary when gene flow is ancient (*T*_GF_ = *T*_DIV_) and large immigration rates (*Nm*> 1) are necessary when gene flow is very recent (*T*_GF_ = *T*_DIV_/10,000) (Figure 4d). We found an exception for *T*_GF_ = *T*_DIV_/10, where both low and high rates lead to uSFS, likely due to the introduction of both ancestral and derived alleles in the population, depending on which allele was fixed ancestral or fixed derived in the sampled population. Interestingly, multimodal SFS only occurs for very specific conditions, that *is* large immigration rates and intermediate duration of gene flow (*T*_GF_ = *T*_DIV_/100, Figure 4d), and in those cases, the internal mode is only seen at high derived frequencies.

### 3.3 Application to human data

**FIGURE 5:**
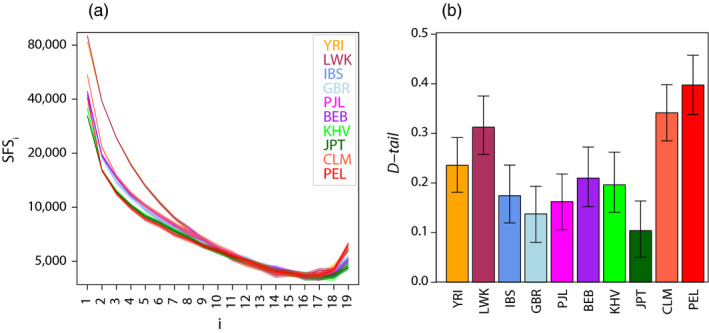
Neutral SFS (a) and associated *D‐tail* statistics (b) observed in ten 1000G human samples. In pane a, ${\text{SFS}}_{i}$ is the number of sites with a derived frequency *i*. In pane b, the whiskers indicate limits of 95% block‐bootstrap confidence intervals

All ten 1000G populations show clear uSFS at neutral sites (Figure 5). Among the four demographic scenarios tested on these human data (Supporting Information 5), only the scenario of genetic *isolation* fails to produce uSFS (Supporting Information 10), shows less good fit especially when looking at normalized SFS (Lapierre, Lambert, & Achaz, 2017), and a significantly lower likelihood than that of the three other scenarios (Supporting Information 11). Expected SFS is found very similar to the observed ones, and the estimated maximum likelihood values are found close to the maximum possible value (computed by assuming that the expected SFS entries would be equal to the observed SFS entries) for the three other scenarios: *isolation with ASM*, *admixture* and *immigration*. Therefore, we cannot distinguish which of these three scenarios is best on the sole basis of their likelihoods. However, we find that an average of 4.38% (2.75% – 7.59%) of ancestral state mis‐assignment is necessary for the *isolation with ASM* model to fit the data. This value is one order of magnitude higher than that previously estimated in Yoruba (0.1%–0.3% in Lapierre, 2017) by using sites for which the nucleotide of an out‐group species is different from the two nucleotides defining a SNP in a focal population (Baudry & Depaulis, 2003). It suggests that ASM in a context of genetic isolation is not the cause of the uSFS observed from the human neutral data, and that one of the two models involving gene flow is a more plausible explanation. Overall, the best parameters inferred from gene flow scenarios generally point to mild and recent gene flow (mean admixture rate *a* = 0.06 and time *T*_ADM_ = 171 generations ago for *admixture* scenario; on average, 72 migrants per generation for 540 generations for the *immigration* scenario, i.e. postlast glacial maximum for non‐African populations).

## 4 DISCUSSION

Gene flow is often overlooked as an explanation for the observation of an excess of high‐frequency‐derived alleles. Natural populations showing uSFS are usually considered as isolated but under selection (Li et al., 2012; Liu et al., 2017; Qanbari & Simianer, 2014; Sabeti et al., 2006). However, this strong assumption of genetic isolation is far from being warranted, as gene flow between populations seems to be the standard in non‐human species (Sexton, Hangartner, & Hoffmann, 2014), sometimes even extending over species boundaries (Shurtliff, 2013; Wang et al., 2019) and persisting despite habitat fragmentation due to human activity (Corlatti, Hacklaänder, Frey‐Roos, HacklÄnder, & Frey‐Roos, 2009). For humans, numerous occurrences of gene flow between populations have been documented at every epochs and on every continent (Hellenthal et al., 2014). Human isolates rather seem to be an exception (Heutink, 2002) and seem to have emerged recently due to geographical and/or cultural barriers, for example populations living on islands or remote places (Roberts, 1976; Serre, Jakobi, & Babron, 1985), or being cultural minorities (Bideau, Brunet, Heyer, & Plauchu, 1994; Capocasa et al., 2013; Mourali‐Chebil & Heyer, 2006), and usually present health and fitness issues (Charlesworth & Willis, 2009; Keller & Waller, 2002; Spielman, Brook, Briscoe, & Frankham, 2004). In this paper, the ten 1000G populations we study all show uSFS in their neutral fraction of genomes, where selection is supposed to have almost no effect (Pouyet et al., 2018), suggesting that they are not genetically isolated populations. More generally, we show with simulations that gene flow alone (i.e. in the absence of any selection, for two very contrasting models of gene flow) can actually easily produce an excess of high‐frequency‐derived alleles and uSFS. Interestingly, we find that uSFS can emerge from gene flow both by (a) the introduction of a few ancestral alleles (*II* and other* IA* models) and (b) by a massive input of derived alleles (during a partial genetic replacement, i.e. *IA* model with admixture rates larger than 0.5). This latter result extends previous ones (Wakeley & Aliacar, 2001), as not only mild gene flow can lead to an excess of high‐frequency‐derived alleles after a single admixture event. Furthermore, for higher rates of gene flow between deeply divergent populations, we manage to simulate W‐shaped SFS, a signal that can also be produced by balancing selection (Bitarello et al., 2018; Croze, Živković, Stephan, & Hutter, 2016), associative overdominance (Gilbert, Pouyet, Excoffier, & Peischl, 2020) or in a heterogeneous structure resulting from divergent sources sampled as a single population (González‐Martínez, Ridout, & Pannell, 2017).

Our results are in line with the fact that human populations are not genetically isolated, even though our study did not formally identify the source of recently incoming lineages. In our models, we used an unsampled or “ghost” population as the source of gene flow (Excoffier et al., 2013), which simply models a reservoir for some divergent lineages now found in the sampled population (Beerli, 2004; Slatkin, 2005). It can represent a population that separated a long time ago from the sampled population, as in the case of a secondary contact after a period of isolation, like in hybrid zones at the population or species level (Alcala, Jensen, Telenti, & Vuilleumier, 2016; Alcala & Vuilleumier, 2014; Hvala & Wood, 2012; Tine et al., 2014). Such hybridization events have occurred repeatedly in human evolution (e.g. between anatomically modern and archaic humans (Dannemann & Racimo, 2018) or after long‐distance dispersals between population of distinct ancestries (Fortes‐Lima et al., 2018; Sedghifar, Brandvain, Ralph, & Coop, 2015; Verdu et al., 2014). Interestingly, if the source population is actually sampled, the joint SFS for the source and the target populations will reveal in the target population an excess of rare or event quite frequent derived alleles, for small and large immigration rates, respectively (Supporting Information 12), as previously reported in population or species having recently reconnected (Alcala et al., 2016; Alcala & Vuilleumier, 2014; Fraïsse et al., 2018; Tellier et al., 2011; Tine et al., 2014). Alternatively, as already mentioned above, the “ghost” population does not need to correspond to a real or an existing population, but can rather simply represent a set of populations surrounding the sampled population, as in large spatially structured populations, which can be described as a continent‐island model (Excoffier, 2004; Hahn, 2018), for example like after a spatial expansion.

This last type of ghost (continent) population can be particularly relevant to model the history of human populations, as we were not able to identify the source of gene flow within the available 1,000 Genomes populations (Supporting Information 13). A consensus scenario for the worldwide expansion of humans is a serial founder effect out of Africa with limited archaic hybridization (Ramachandran et al., 2005; Stringer, 2014). As uSFS has actually been observed in simulations of range expansions, one could think that gene surfing having occurred during past human range expansions could explain the observed uSFS (Sousa et al., 2014). However, during human expansions, both recurrent founder effects at the front and migration between neighbouring demes in the wake of the front certainly occurred, such that gene surfing at the front could have promoted the fixation of different alleles in different sectors and a mixing of these sectors in the wake of the expansion could have led to uSFS (Peischl, Dupanloup, Bosshard, & Excoffier, 2016). We have run additional simulations to investigate the impact of gene flow during range expansions on the SFS (Supporting Information 14). We find that uSFS is only observed when gene flow between adjacent populations on the front is associated with the expansion, showing that gene surfing alone cannot lead to an excess of high‐frequency‐derived alleles. In addition, we find that uSFS can also stem from a Wahlund effect, that *is* when the SFS is computed from a population with hidden subdivisions (Supporting Information 15). Therefore, uSFS can emerge from naturally occurring gene flow or from artefactual structure resulting from the sampling of divergent lineages, as both will result in the potential mixing of differentially fixed alleles.

Whereas uSFS is never observed in completely isolated populations under a classical Kingman coalescent model, they can certainly exist under multiple‐merger coalescent (MMC) models (Eldon et al., 2015; Pitman, 1999; Sagitov, 1999; Sargsyan & Wakeley, 2008; Schweinsberg, 2000; Tellier & Lemaire, 2014), which occur under recurrent episodes of selective sweeps or for extremely skewed distributions of offspring numbers (e.g. oyster, cod, bacteria and viruses (Árnason & Halldoŕsdóttir, 2015; Sargsyan & Wakeley, 2008; Tellier & Lemaire, 2014)). Since different parts of the genome can be differentially affected by selection, a mixture of classical and multiple‐merger coalescent models could be used to model whole genomes (Rice et al., 2018). Contrastingly, gene flow into a population should affect the whole genome, even though effective migration rates may be affected by intragenomic selective processes as well (Petry, 1983; Sousa & Hey, 2013). It would therefore be interesting to include the effect of gene flow in the context of multiple‐merger models as well. Along the same lines, procedure contrasting the SFS at different positions of the genome to evidence selection (Fay & Wu, 2000; Kim & Stephan, 2002; Nielsen et al., 2005; Pavlidis et al., 2010; Zeng, Fu, Shi, & Wu, 2006) or methods using the SFS to infer the distribution of fitness effects (Eyre‐Walker & Keightley, 2007; Kim, Huber, & Lohmueller, 2017; Tataru, Mollion, Glémin, & Bataillon, 2017) do not take gene flow into account and could thus lead to biased inferences. We therefore hope that our study would promote the inclusion of gene flow when studying the effect of selection on genomic diversity.

## CONFLICT OF INTEREST

None declared.

## AUTHOR CONTRIBUTIONS

L.E. and N.M. designed the study and wrote the manuscript, L.E. performed *fastsimcoal2* parameter estimations from 1000Genomes data, N.M. run the simulations and analysed the results.

## Supporting information

Supplementary Material

Supplementary Material

Supplementary Material

Supplementary Material

Supplementary Material

Supplementary Material

Supplementary Material

Supplementary Material

Supplementary Material

Supplementary Material

Supplementary Material

Supplementary Material

Supplementary Material

Supplementary Material

Supplementary Material

## Data Availability

SFS from the simulations and for the ten studied 1,000 Genomes populations is available upon request to the authors. Furthermore, the input files necessary to repeat the simulations are provided in the Supplementary Information.
