# Supplementary material for: Gene flow as a simple cause for an excess of high‐frequency‐derived alleles
Source: Evol Appl. 2020 Jun 2;13(9):2254–63. doi: 10.1111/eva.12998 (PMC7513730; doi:10.1111/eva.12998)

**Supp.** **Information** **1** **–** **Effect of gene flow on SFS properties for *N* = 40,000.** *D-tail* (left pane) and shapes of SFS (right pane) obtained *n =*10 under different conditions: A&B) *IA* model with $\tau_{ADM}=0$ for different $\tau_{DIV}$ and *a*; C&D) *IA* model with $\tau_{DIV}=2.5$ for different $\tau_{ADM}$ and *a*; E&F) *II* model with $\tau_{DIV}=2.5$ for different *T_GF_* and *Nm*. Note that we performed boot-straps; however, the 95% block-bootstrap confidence intervals are so small that there are hidden by the solid line and dots on the left figures.


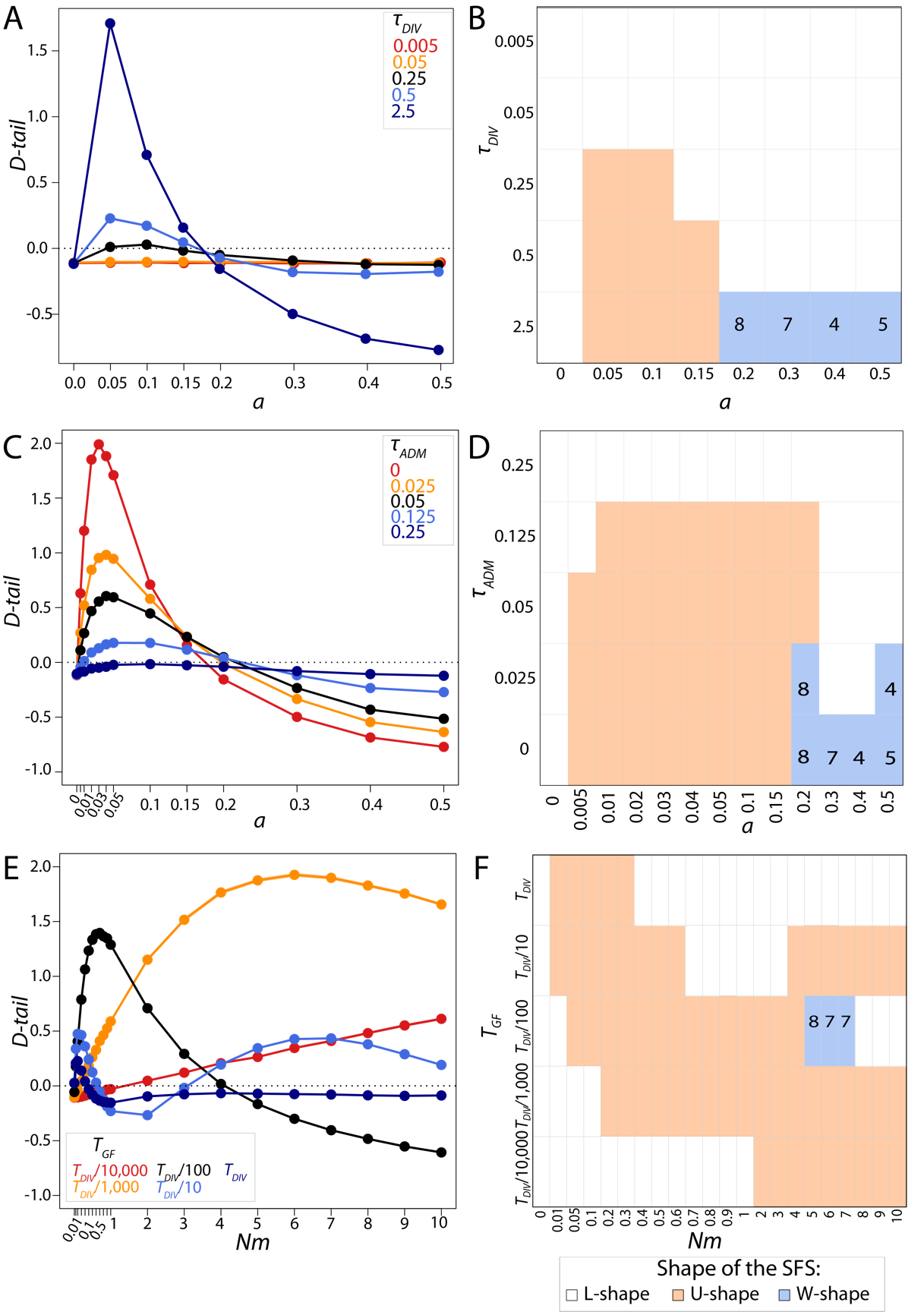

Supplement: Supplementary file 1 — Supplementary Material [file EVA-13-2254-s001.docx]
