# Supplementary material for: Gene flow as a simple cause for an excess of high‐frequency‐derived alleles
Source: Evol Appl. 2020 Jun 2;13(9):2254–63. doi: 10.1111/eva.12998 (PMC7513730; doi:10.1111/eva.12998)

**Supp.** **Information** **2** **–** **Effect of explicit linkage between SNPs** for SFS simulated under an *IA* model, with *n* = 10, $\tau_{DIV}=2.5$, $\tau_{ADM}=0$ for different *a* values. In dashed lines, SFS obtained from 100 Mb modelled as 10,000 blocks of 1,000 independent non-recombining regions of 100 bp; solid lines, SFS obtained from 100 Mb modelled as 10,000 independent blocks of 100,00bp with a recombination rate per block of 10^-8^/bp/generation; semi-transparent colors define 95% block-bootstrap confidence intervals. $SFS_{i}$is the number of sites with a derived frequency *i.*


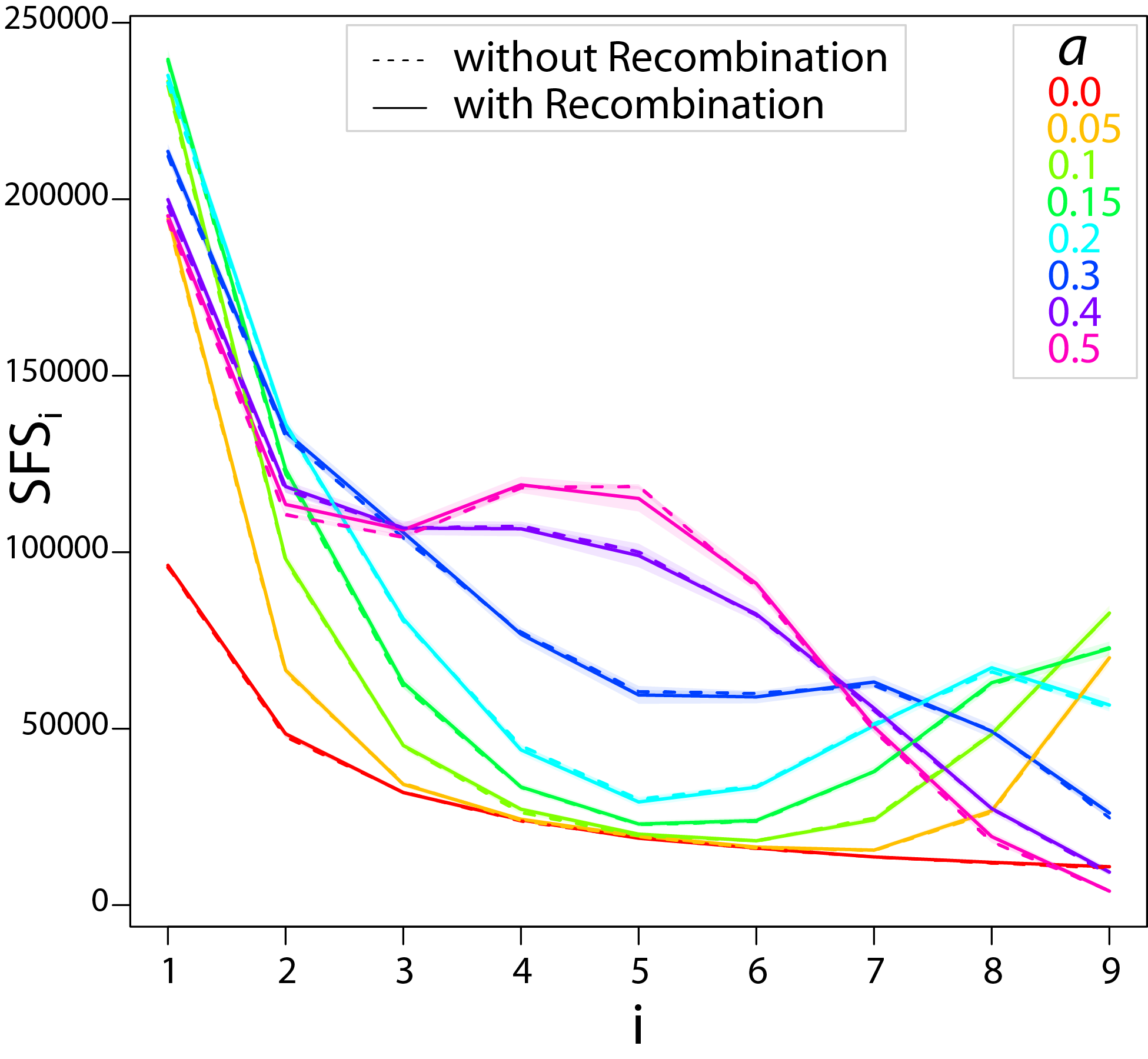

Supplement: Supplementary file 2 — Supplementary Material [file EVA-13-2254-s002.docx]
