# Supplementary material for: Gene flow as a simple cause for an excess of high‐frequency‐derived alleles
Source: Evol Appl. 2020 Jun 2;13(9):2254–63. doi: 10.1111/eva.12998 (PMC7513730; doi:10.1111/eva.12998)
Supplement: Supplementary file 3 — Supplementary Material [file EVA-13-2254-s003.docx]

**Supp. Information 3 –** *fastsimcoal2* generic input file used to perform IA and II simulations.

**IA.par**

//Parameters for the coalescence simulation program : fsc3605.exe

2

//Population effective sizes (number of genes)

NPop

NPop

//Samples sizes

0 0 0

nSamp 0 0

//Growth rates : negative growth implies population expansion

0

0

//Number of migration matrices : 0 implies no migration between demes

0

//historical event: time, source, sink, migrants, new deme size, new growth rate, migration matrix index

2 historical event

TAdm 1 0 admRate 1 0 0

TDiv 1 0 1 1 0 0

//Number of independent loci [chromosome]

1000 0

//Per chromosome: Number of contiguous linkage Block: a block is a set of contiguous loci

1

//per Block:data type, number of loci, per generation recombination and mutation rates and optional parameters

DNA 100 0 1.20e-8 OUTEXP

**II.par**

//Parameters for the coalescence simulation program : fsc26.exe

2

//Population effective sizes (number of genes)

NPop

NPop

//Samples sizes

0 0 0

nSamp 0 0

//Growth rates : negative growth implies population expansion

0

0

//Number of migration matrices : 0 implies no migration between demes

2

//Migrmat 0

0 0

migrRate 0

//Migrmat 1

0 0

0 0

//historical event: time, source, sink, migrants, new deme size, new growth rate, migration matrix index

2 historical event

TGf 0 0 0 1 0 1

TDiv 1 0 1 1 0 1

//Number of independent loci [chromosome]

1000 0

//Per chromosome: Number of contiguous linkage Block: a block is a set of contiguous loci

1

//per Block:data type, number of loci, per generation recombination and mutation rates and optional parameters

DNA 100 0 1.20e-8 OUTEXP
