# Supplementary material for: Gene flow as a simple cause for an excess of high‐frequency‐derived alleles
Source: Evol Appl. 2020 Jun 2;13(9):2254–63. doi: 10.1111/eva.12998 (PMC7513730; doi:10.1111/eva.12998)

Supp. Information 4 – SFS obtained for all tested conditions with n = 10 haploid individuals and N = 4,000, where i is the derived allele frequency. Dots and solid lines were obtained from simulated data sets, and semi-transparent colors define 95% block-bootstrap confidence intervals (note that these confidence intervals are so small that they are barely visible on these figures) .

IA model: $\tau_{ADM} = 0; N = 4{,}000\ ; a \leq 0.5$

$\tau_{DIV} = 0.005$

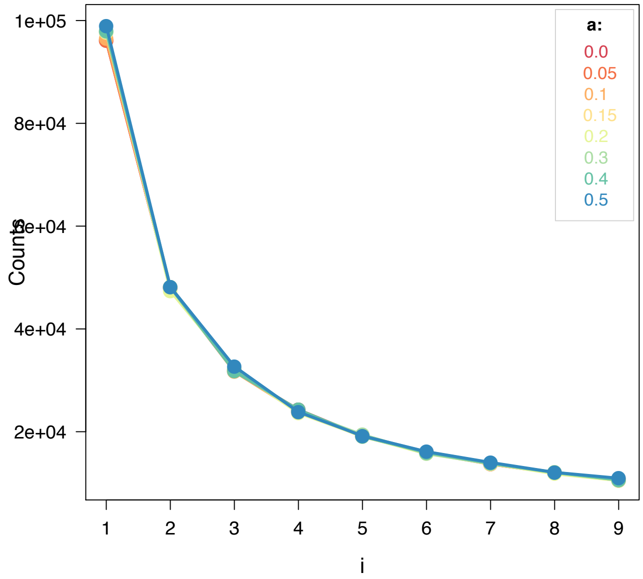

$\tau_{DIV} = 0.05$

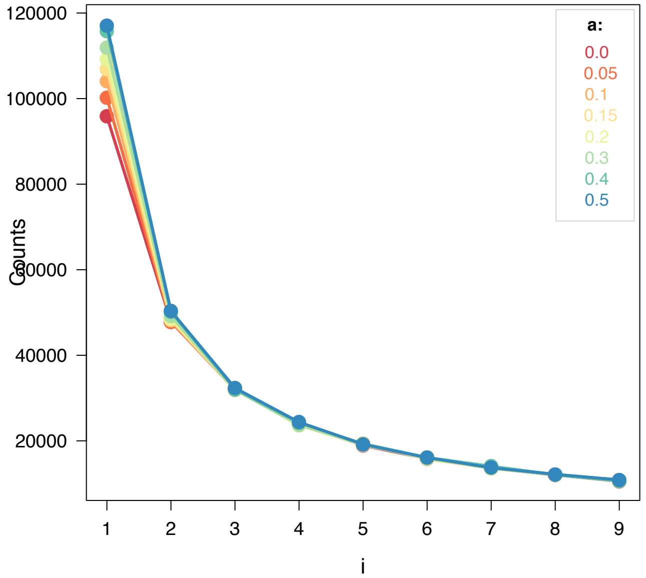

$\tau_{DIV} = 0.25$

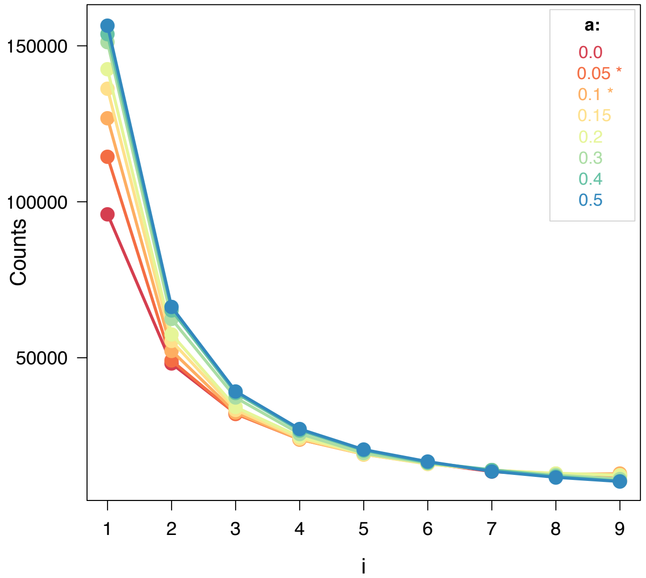

$\tau_{DIV} = 0.5$

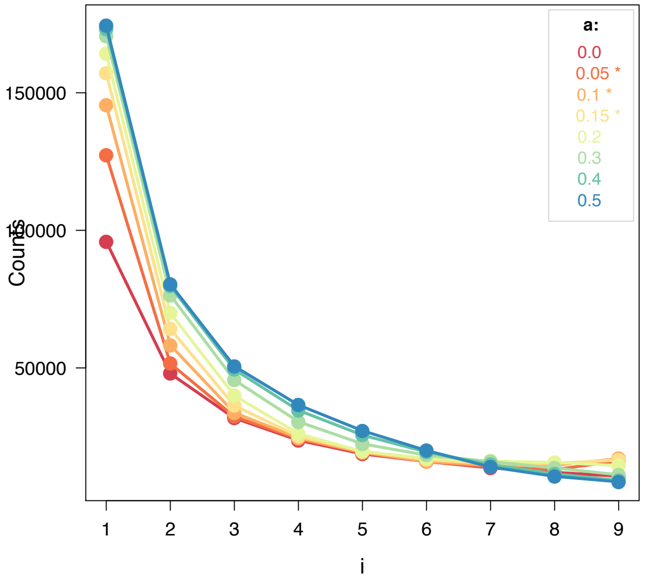

$\tau_{DIV} = 2.5$

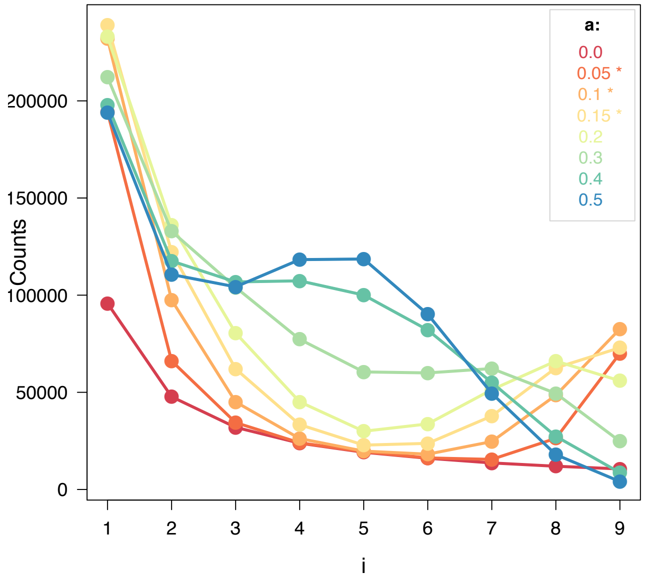

***IA* model:** $\tau_{ADM} = 0$; $N = 4{,}000$; $a > 0.5$

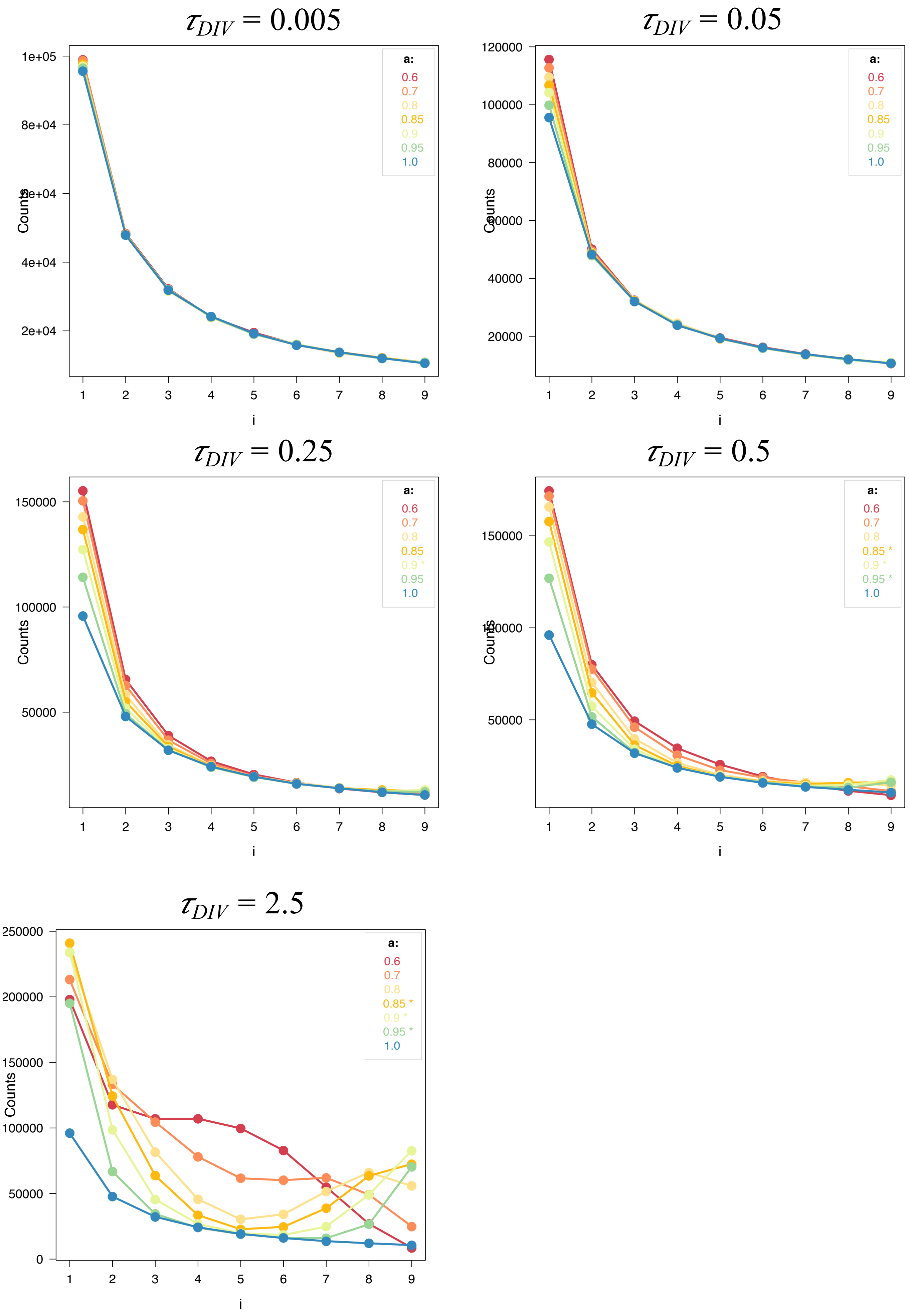

***IA* model:** τ_{DIV} **= 2.5; *N* = 4,000 ; *a* ≤ 0.5**

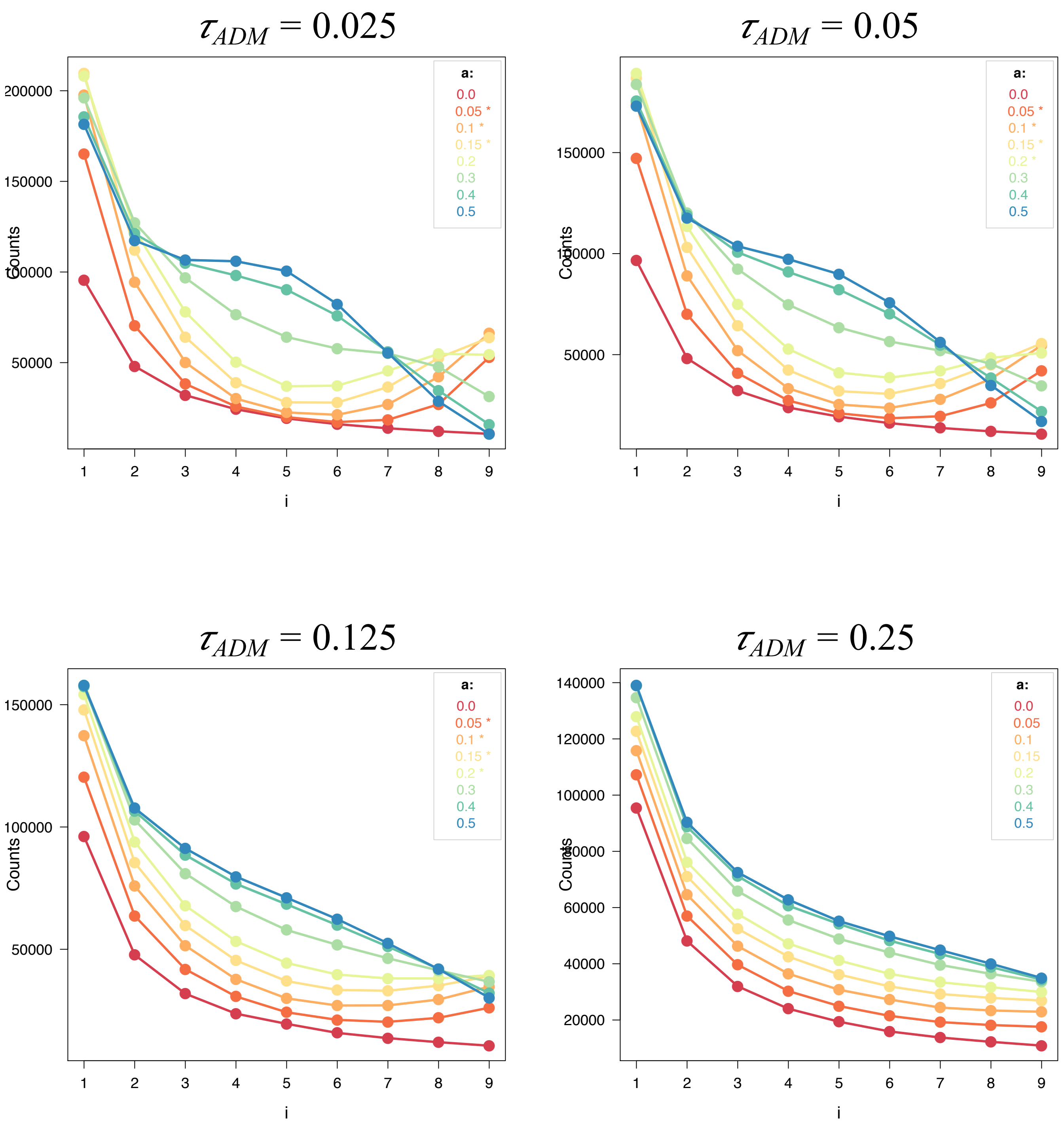

***II* model:** $\tau_{DIV} = T_{DIV}/N = 2.5$; $N = 4{,}000$

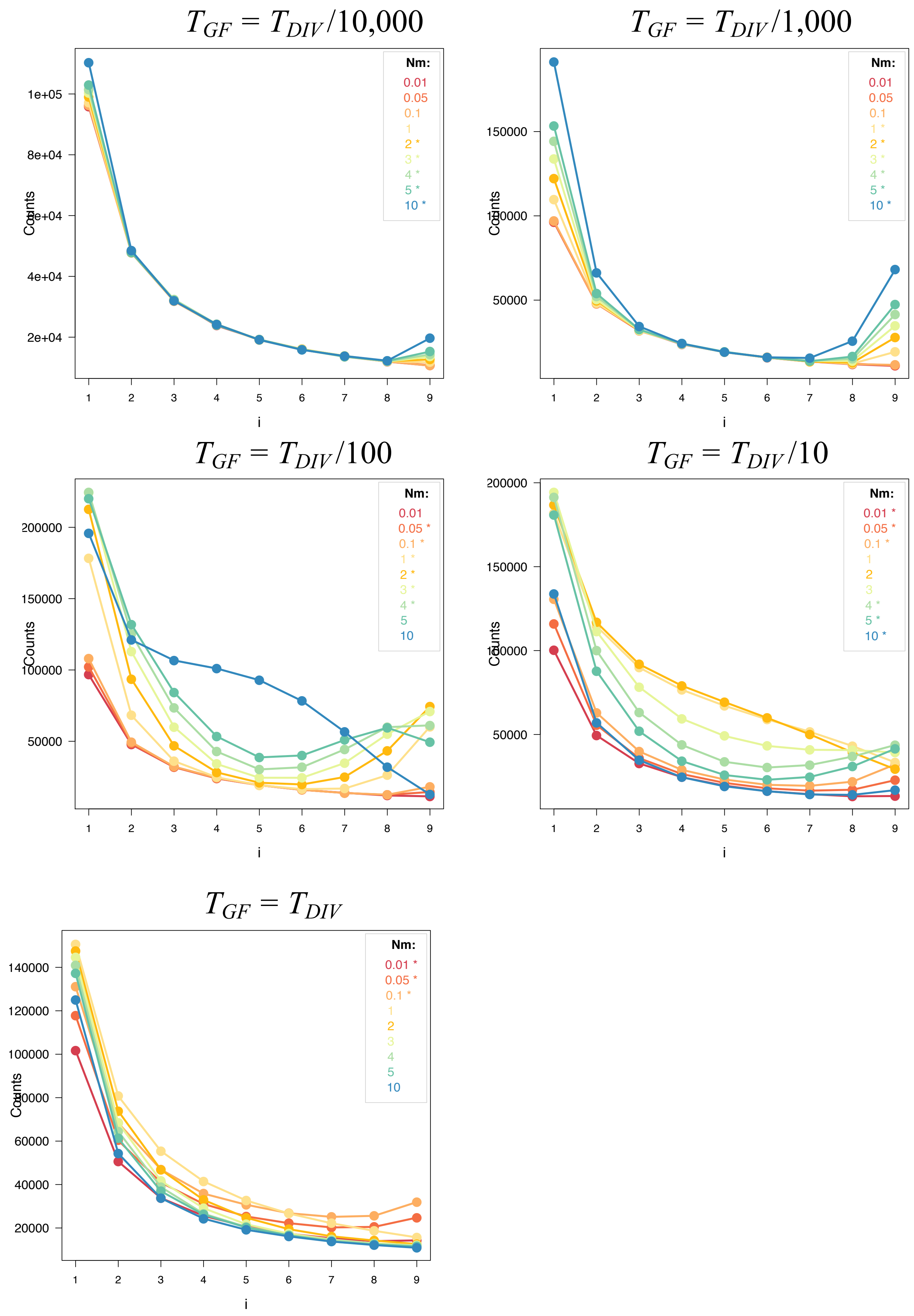

Supplement: Supplementary file 4 — Supplementary Material [file EVA-13-2254-s004.pdf]
