# Supplementary material for: Gene flow as a simple cause for an excess of high‐frequency‐derived alleles
Source: Evol Appl. 2020 Jun 2;13(9):2254–63. doi: 10.1111/eva.12998 (PMC7513730; doi:10.1111/eva.12998)

**Supp.** **Information** **5** **–** Scenarios designed to test the cause of uSFS observed in ten populations of 10 diploid individuals from the 1000Genomes panel: A) genetic isolation; or gene flow from a ghost population of haploid size 20,000; B) immigration at rate *m* since the most recent bottleneck event or C) admixture at rate *a* at *TAdm*. Based on Pouyet *et al.* (2018), the studied population comes from an ancestral population of fixed size *NANC*= 40,000 haploids and underwent three bottlenecks lasting for 100 generations at *TBot_i_* with a size *NBOT_i_* that led to population sizes (*NPAST*, *NPREV* and *NCUR*).


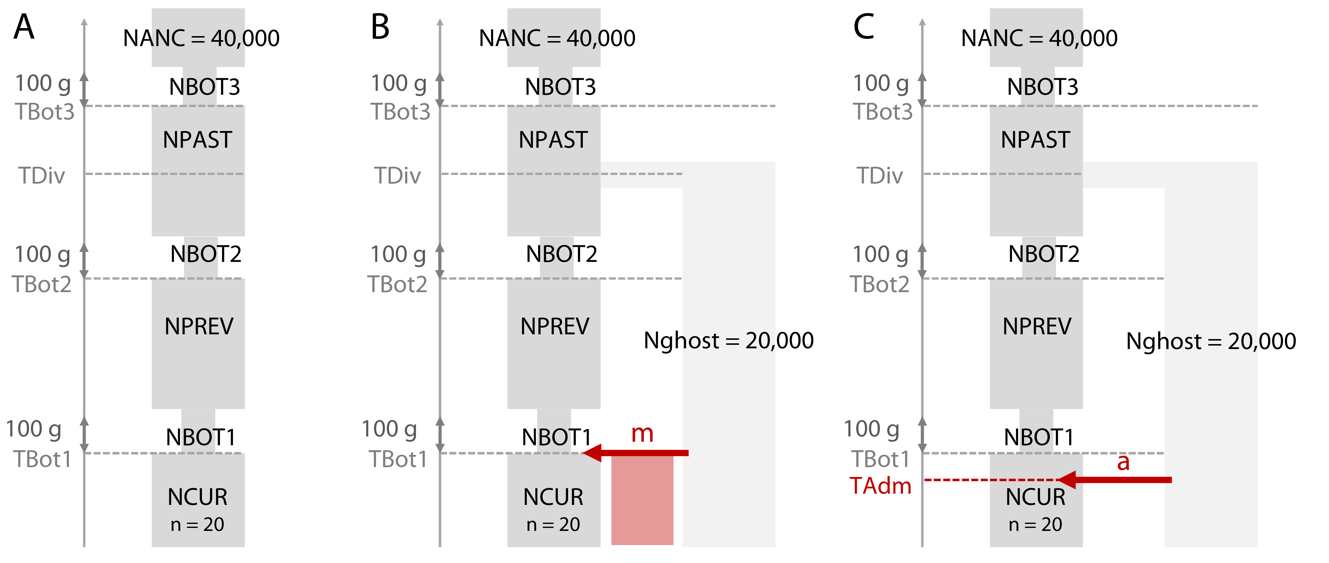

Supplement: Supplementary file 5 — Supplementary Material [file EVA-13-2254-s005.docx]
