# Supplementary material for: Gene flow as a simple cause for an excess of high‐frequency‐derived alleles
Source: Evol Appl. 2020 Jun 2;13(9):2254–63. doi: 10.1111/eva.12998 (PMC7513730; doi:10.1111/eva.12998)
Supplement: Supplementary file 6 — Supplementary Material [file EVA-13-2254-s006.docx]

**Supp. Information 6 –** *fastsimcoal2* generic input files used to estimate parameters

for each of the 1000Genomes populations and scenarios shown in Supp. Information 5.

**GENETIC ISOLATION MODEL**

**POP.tpl**

//Parameters for the coalescence simulation program : simcoal.exe

2 samples to simulate :

//Population effective sizes (number of genes)

NCUR

20000

//Samples sizes and samples age

20

0

//Growth rates: negative growth implies population expansion

0

0

//Number of migration matrices : 0 implies no migration between demes

0

//historical event: time, source, sink, migrants, new deme size, growth rate, migr mat index

6 historical event

TBot1 0 0 0 RESBot1 0 0

TEndBot1 0 0 0 RESEndBot1 0 0

TBot2 0 0 0 RESBot2 0 0

TEndBot2 0 0 0 RESEndBot2 0 0

TAncBot 0 0 0 RESAncBot 0 0

TEndAncBot 0 0 0 RESEndAncBot 0 0

//Number of independent loci [chromosome]

1 0

//Per chromosome: Number of contiguous linkage Block: a block is a set of contiguous loci

1

//per Block:data type, number of loci, per gen recomb and mut rates

FREQ 1 0 1.25e-8 OUTEXP

**POP.est**

// Priors and rules file

// *********************

[PARAMETERS]

//#isInt? #name #dist.#min #max

//all N are in number of haploid individuals

//1 NANC unif 100 1000000 output

1 NCUR unif 100 1000000 output

1 NBOT1 unif 5 10000 output

1 NPREV unif 100 100000 output

1 NBOT2 unif 5 10000 output

1 NPAST unif 10 100000 output

1 NBOT3 unif 10 10000 output

1 TBot1 unif 10 1000 output

1 TToBot2 unif 10 10000 hide

1 TToBot3 unif 10 10000 hide

//1 TAdm unif 10 5000 output

//0 padm unif 0 0.1 output

//1 TPlusDiv unif 10 5000 hide

[RULES]

[COMPLEX PARAMETERS]

//NANC is assumed to be 40000, which is in between these two estimates from Malaspinas et al. 2016 (X2)

//Ne ancestral modern humans 23,275

//Ne ancestral archaics/humans 18,296

0 RESBot1 = NBOT1/NCUR hide

0 RESEndBot1 = NPREV/NBOT1 hide

0 RESBot2 = NBOT2/NPREV hide

0 RESEndBot2 = NPAST/NBOT2 hide

0 RESAncBot = NBOT3/NPAST hide

0 RESEndAncBot = 40000/NBOT3 hide

1 TEndBot1 = TBot1+100 hide

1 TBot2 = TEndBot1+TToBot2 output

1 TEndBot2 = TBot2+100 hide

1 TAncBot = TEndBot2+TToBot3 output

1 TEndAncBot = TAncBot+100 hide

//1 TDiv = TAdm+TPlusDiv output

**ISOLATION WITH IMMIGRATION MODEL**

**POP.tpl**

//Parameters for the coalescence simulation program : simcoal.exe

2 samples to simulate :

//Population effective sizes (number of genes)

NCUR

20000

//Samples sizes and samples age

20

0

//Growth rates: negative growth implies population expansion

0

0

//Number of migration matrices : 0 implies no migration between demes

2

// Matrix 0

0 m_0_1

0 0

// Matrix 1

0 0

0 0

//historical event: time, source, sink, migrants, new deme size, growth rate, migr mat index

7 historical events

TBot1 0 0 0 RESBot1 0 1

TEndBot1 0 0 0 RESEndBot1 0 1

TBot2 0 0 0 RESBot2 0 1

TEndBot2 0 0 0 RESEndBot2 0 1

TAncBot 0 0 0 RESAncBot 0 1

TEndAncBot 0 0 0 RESEndAncBot 0 1

TDiv 1 0 1 1 0 1

//Number of independent loci [chromosome]

1 0

//Per chromosome: Number of contiguous linkage Block: a block is a set of contiguous loci

1

//per Block:data type, number of loci, per gen recomb and mut rates

FREQ 1 0 1.25e-8 OUTEXP

**POP.est**

// Priors and rules file

// *********************

[PARAMETERS]

//#isInt? #name #dist.#min #max

//all N are in number of haploid individuals

//1 NANC unif 100 1000000 output

1 NCUR unif 100 1000000 output

1 NBOT1 unif 5 10000 output

1 NPREV unif 100 100000 output

1 NBOT2 unif 5 10000 output

1 NPAST unif 10 100000 output

1 NBOT3 unif 10 10000 output

1 TBot1 unif 10 1000 output

1 TToBot2 unif 10 10000 hide

1 TToBot3 unif 10 10000 hide

1 TDiv unif 10 10000 output

0 Nm unif 0 100 output

[RULES]

[COMPLEX PARAMETERS]

//NANC is assumed to be 40000, which is in between these two estimates from Malaspinas et al. 2016 (X2)

//Ne ancestral modern humans 23,275

//Ne ancestral archaics/humans 18,296

0 RESBot1 = NBOT1/NCUR hide

0 RESEndBot1 = NPREV/NBOT1 hide

0 RESBot2 = NBOT2/NPREV hide

0 RESEndBot2 = NPAST/NBOT2 hide

0 RESAncBot = NBOT3/NPAST hide

0 RESEndAncBot = 40000/NBOT3 hide

1 TEndBot1 = TBot1+100 hide

1 TBot2 = TEndBot1+TToBot2 output

1 TEndBot2 = TBot2+100 hide

1 TAncBot = TEndBot2+TToBot3 output

1 TEndAncBot = TAncBot+100 hide

0 m_0_1 = Nm/NCUR output

**ISOLATION WITH ADMIXTURE MODEL**

**POP.tpl**

//Parameters for the coalescence simulation program : simcoal.exe

2 samples to simulate :

//Population effective sizes (number of genes)

NCUR

20000

//Samples sizes and samples age

20

0

//Growth rates: negative growth implies population expansion

0

0

//Number of migration matrices : 0 implies no migration between demes

0

//historical event: time, source, sink, migrants, new deme size, growth rate, migr mat index

8 historical event

TBot1 0 0 0 RESBot1 0 0

TEndBot1 0 0 0 RESEndBot1 0 0

TBot2 0 0 0 RESBot2 0 0

TEndBot2 0 0 0 RESEndBot2 0 0

TAncBot 0 0 0 RESAncBot 0 0

TEndAncBot 0 0 0 RESEndAncBot 0 0

TAdm 0 1 padm 1 0 0

TDiv 1 0 1 1 0 0

//Number of independent loci [chromosome]

1 0

//Per chromosome: Number of contiguous linkage Block: a block is a set of contiguous loci

1

//per Block:data type, number of loci, per gen recomb and mut rates

FREQ 1 0 1.25e-8 OUTEXP

**POP.est**

// Priors and rules file

// *********************

[PARAMETERS]

//#isInt? #name #dist.#min #max

//all N are in number of haploid individuals

//1 NANC unif 100 1000000 output

1 NCUR unif 100 1000000 output

1 NBOT1 unif 5 10000 output

1 NPREV unif 100 100000 output

1 NBOT2 unif 5 10000 output

1 NPAST unif 10 100000 output

1 NBOT3 unif 10 10000 output

1 TBot1 unif 10 1000 output

1 TToBot2 unif 10 10000 hide

1 TToBot3 unif 10 10000 hide

1 TAdm unif 10 5000 output

0 padm unif 0 0.1 output

1 TPlusDiv unif 10 5000 hide

[RULES]

[COMPLEX PARAMETERS]

//NANC is assumed to be 40000, which is in between these two estimates from Malaspinas et al. 2016 (X2)

//Ne ancestral modern humans 23,275

//Ne ancestral archaics/humans 18,296

0 RESBot1 = NBOT1/NCUR hide

0 RESEndBot1 = NPREV/NBOT1 hide

0 RESBot2 = NBOT2/NPREV hide

0 RESEndBot2 = NPAST/NBOT2 hide

0 RESAncBot = NBOT3/NPAST hide

0 RESEndAncBot = 40000/NBOT3 hide

1 TEndBot1 = TBot1+100 hide

1 TBot2 = TEndBot1+TToBot2 output

1 TEndBot2 = TBot2+100 hide

1 TAncBot = TEndBot2+TToBot3 output

1 TEndAncBot = TAncBot+100 hide

1 TDiv = TAdm+TPlusDiv output
