# Supplementary material for: Gene flow as a simple cause for an excess of high‐frequency‐derived alleles
Source: Evol Appl. 2020 Jun 2;13(9):2254–63. doi: 10.1111/eva.12998 (PMC7513730; doi:10.1111/eva.12998)
Supplement: Supplementary file 7 — Supplementary Material [file EVA-13-2254-s007.docx]

**Supp.** **Information** **7** **–** Shape of the observed SFS simulated under *IA* scenarios with different admixture rates *a* when the admixture event occurred 0 generations ago. Black numbers indicate the derived frequency *i* of the internal mode of W-shaped SFS.

***
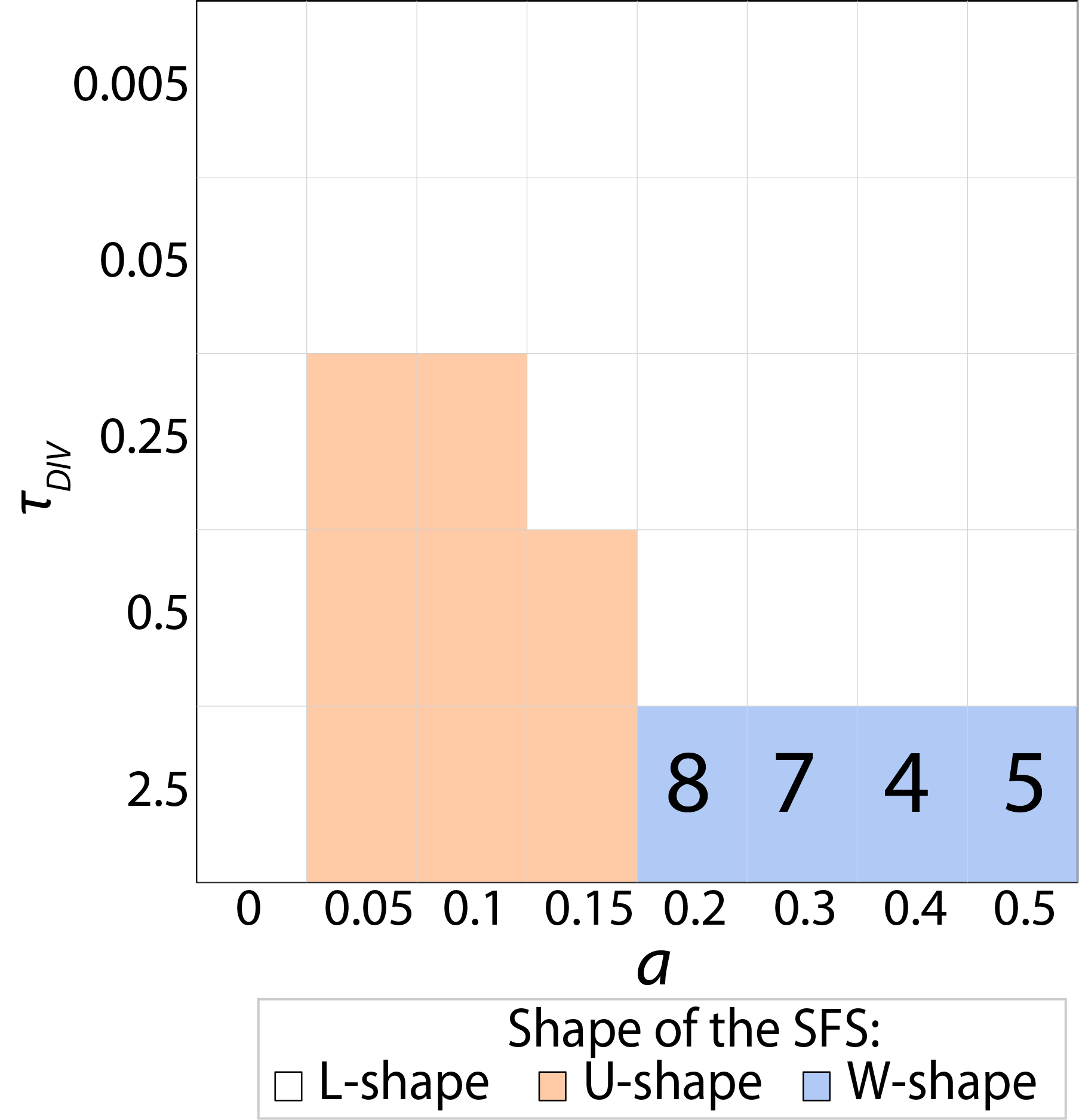
***
