# Supplementary material for: Gene flow as a simple cause for an excess of high‐frequency‐derived alleles
Source: Evol Appl. 2020 Jun 2;13(9):2254–63. doi: 10.1111/eva.12998 (PMC7513730; doi:10.1111/eva.12998)
Supplement: Supplementary file 8 — Supplementary Material [file EVA-13-2254-s008.docx]

**Supp.** **Information** **8** **–** **Effect of large sample size on SFS properties** for SFS simulated under an *IA* model, with *n* = 50, $\tau_{DIV}=2.5$, $\tau_{ADM}=0$ for different *a* values. In dashed lines, uSFSs; solid lines, W-shaped SFSs (or L-shaped for *a*= 0). $SFS_{i}$is the number of sites with a derived frequency *i.*

***
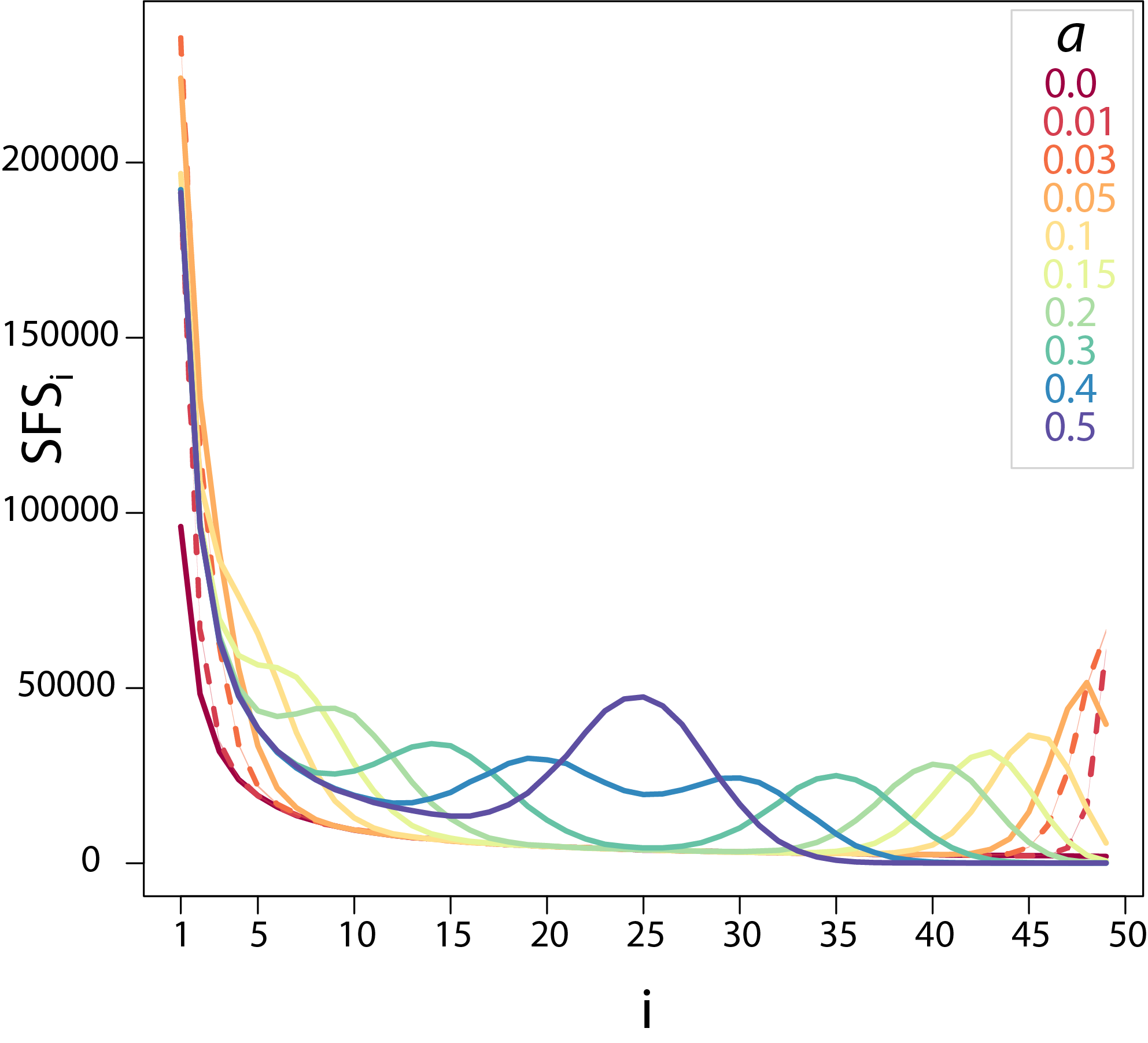
***
