# Supplementary material for: Gene flow as a simple cause for an excess of high‐frequency‐derived alleles
Source: Evol Appl. 2020 Jun 2;13(9):2254–63. doi: 10.1111/eva.12998 (PMC7513730; doi:10.1111/eva.12998)
Supplement: Supplementary file 9 — Supplementary Material [file EVA-13-2254-s009.docx]

**Supp.** **Information** **9 – Effect of partial replacement scenario on SFS properties.** Summary statistics resulting from simulations under an *IA* scenario for *n* = 10 with different partial replacement rates *a* when the admixture event occurred 0 generations ago.

A) *D-tail* statistic for various $\tau_{DIV}$ values; B) for $\tau_{DIV}=2.5$, proportion of loci in the sampled population that were fixed-ancestral before the admixture event and which show *i* derived alleles afterwards.

In pane A, dots and solid lines were obtained from simulated data sets, and semi-transparent colors define 95% block-bootstrap confidence intervals. In pane B, dashed lines stand for uSFS and solid lines stand for W-shaped SFS.

***
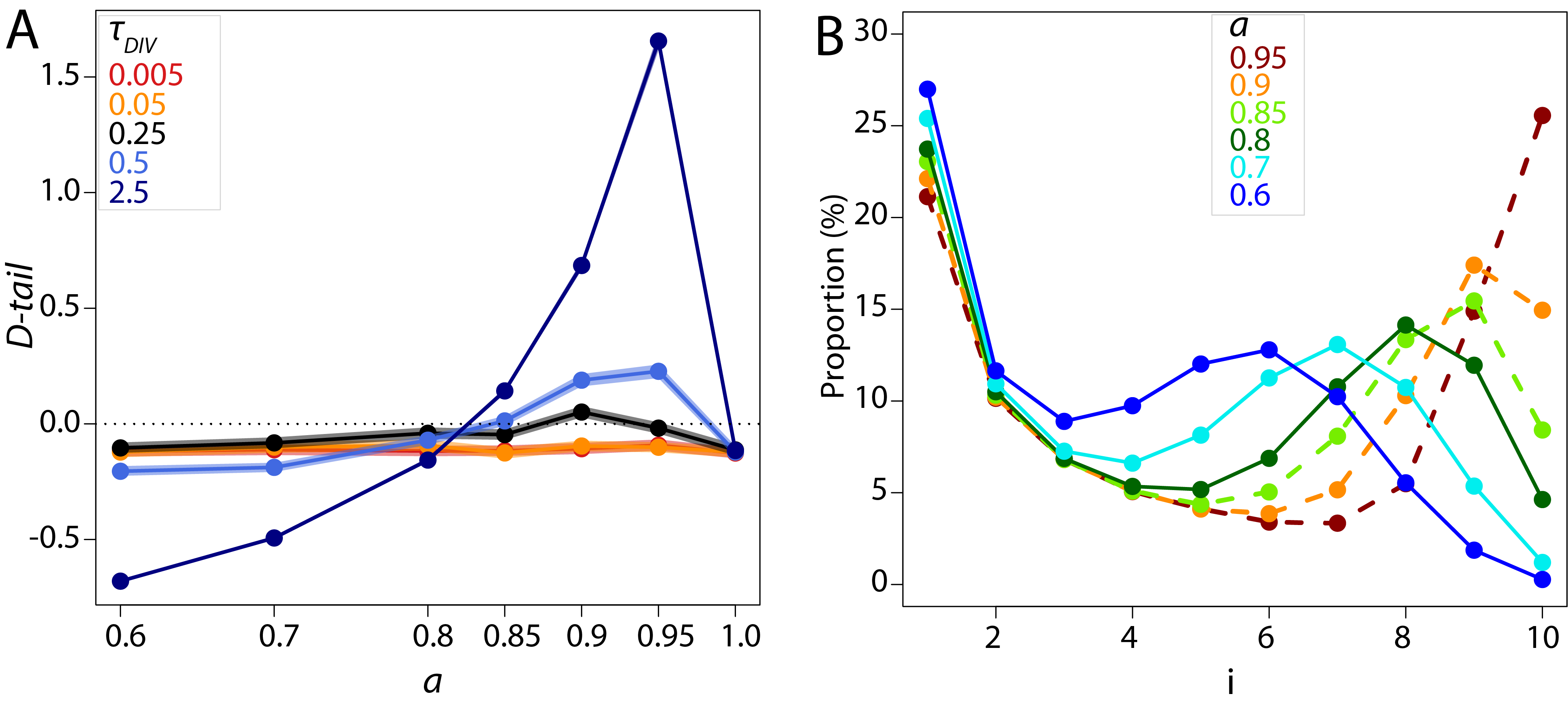
***
