# Supplementary material for: Gene flow as a simple cause for an excess of high‐frequency‐derived alleles
Source: Evol Appl. 2020 Jun 2;13(9):2254–63. doi: 10.1111/eva.12998 (PMC7513730; doi:10.1111/eva.12998)
Supplement: Supplementary file 10 — Supplementary Material [file EVA-13-2254-s010.pdf]

Supp. Information 10 – Fit of the SFS observed for each of the 1000G populations and simulated under the four tested scenarios shown in Supp. Info 5. Top panel corresponds to unfolded SFS where SFS[i] is the number of sites with a derived frequency i. Bottom panel corresponds to normalized SFS where each entry was divided by its expectation in a stationary population (Lapierre *et al.*, 2017).

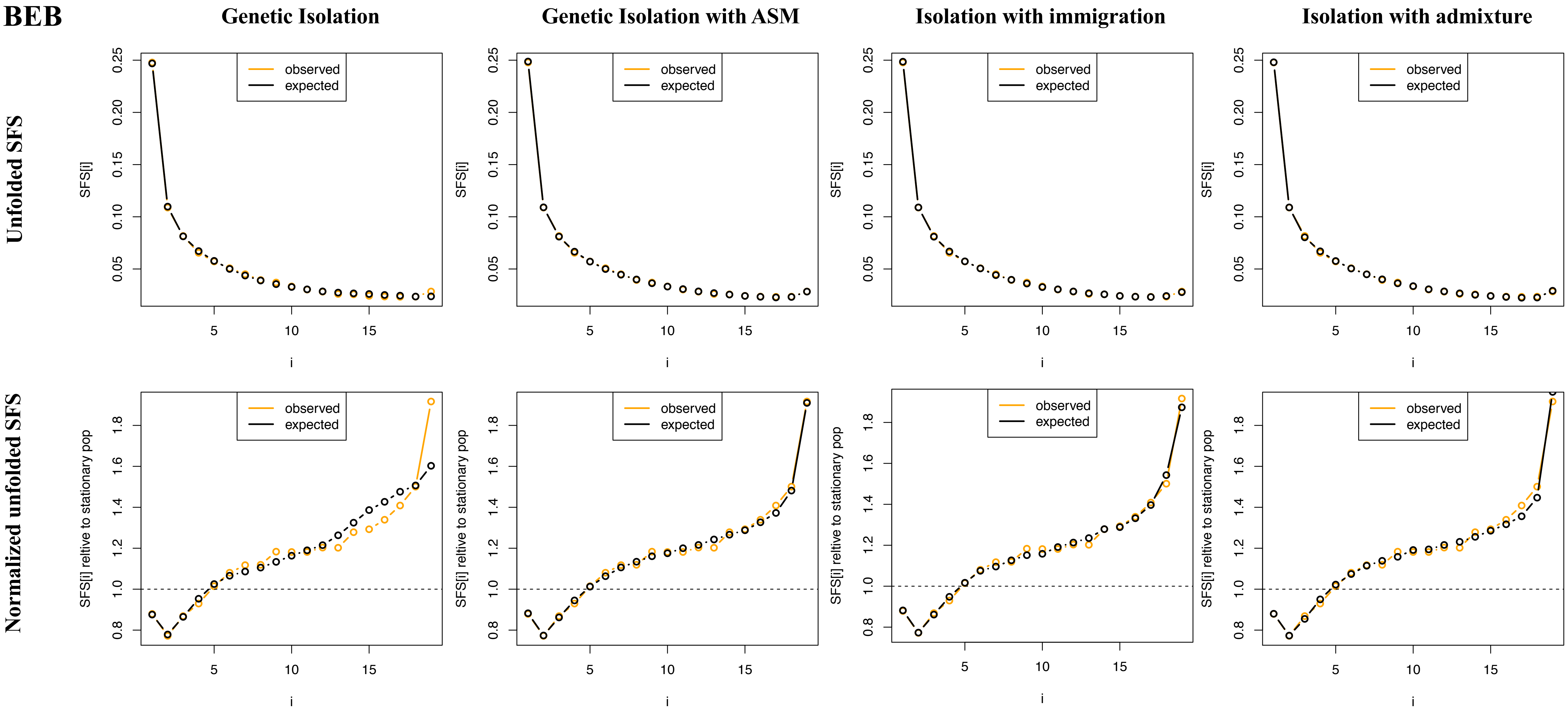

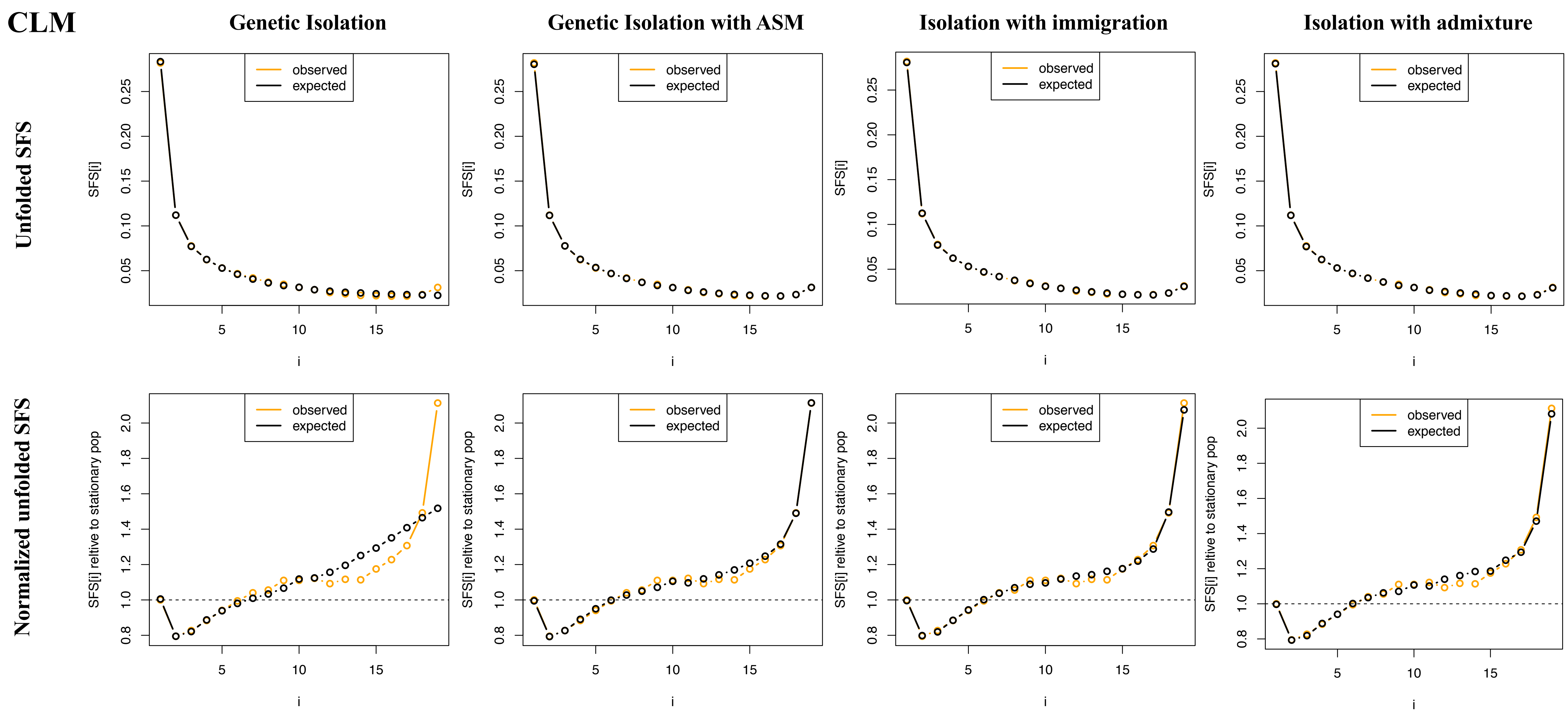

CLM
Genetic Isolation
Genetic Isolation with ASM
Isolation with immigration
Isolation with admixture
Unfolded SFS
Normalized unfolded SFS
observed
expected
SFS[i]
SFS[i] reltive to stationary pop
i

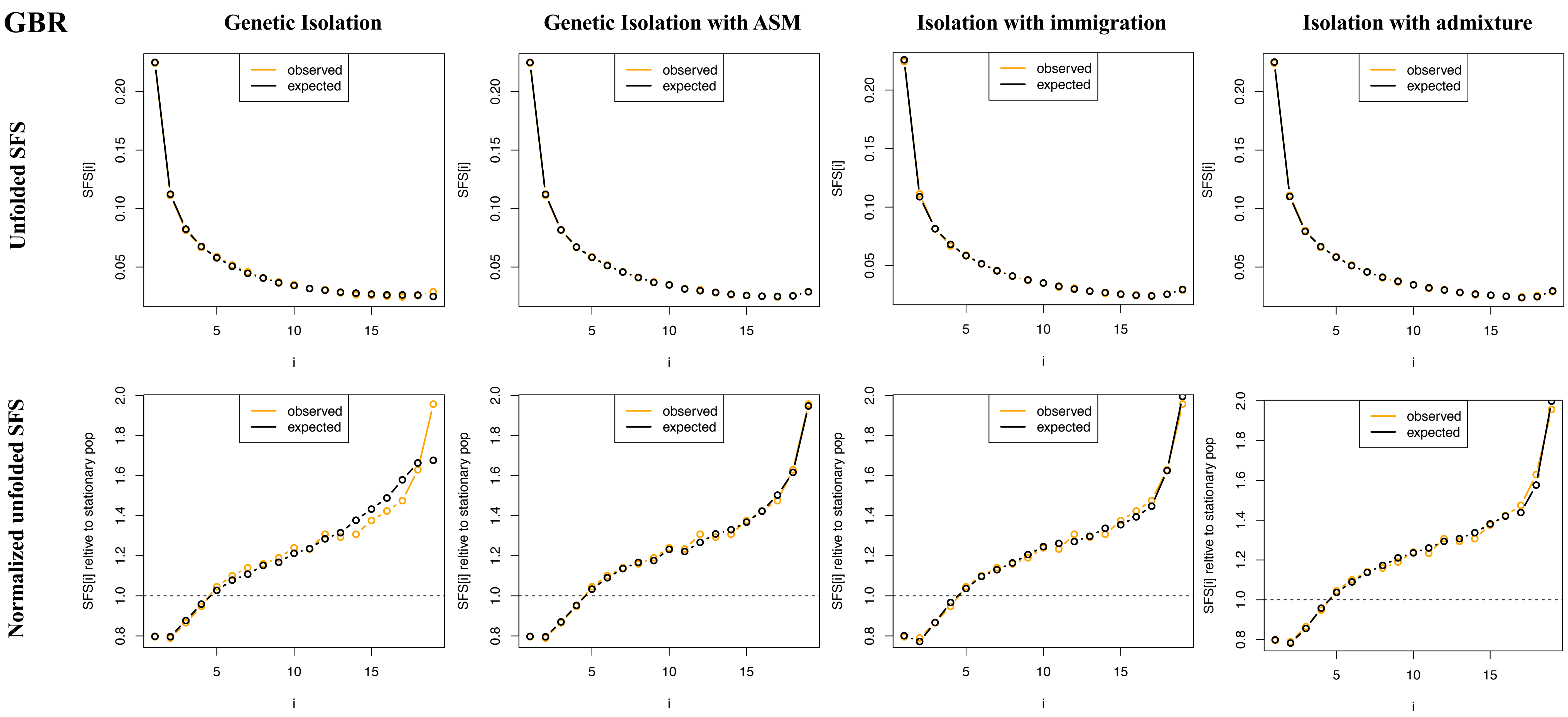

GBR
Genetic Isolation
Genetic Isolation with ASM
Isolation with immigration
Isolation with admixture
Unfolded SFS
Normalized unfolded SFS
observed
expected
SFS[i]
SFS[i] reltive to stationary pop
i

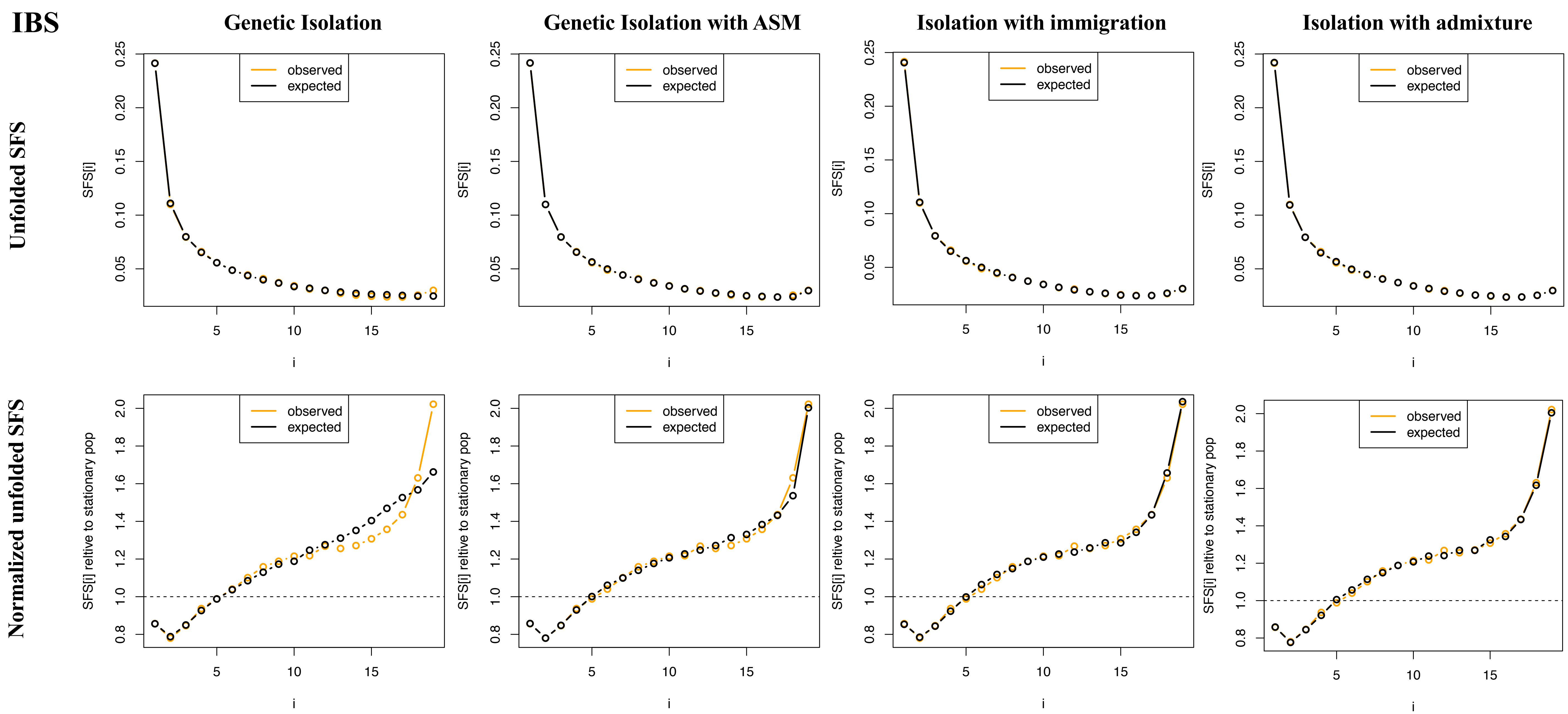

IBS
Genetic Isolation
Genetic Isolation with ASM
Isolation with immigration
Isolation with admixture
Unfolded SFS
Normalized unfolded SFS
observed
expected
SFS[i]
SFS[i] reltive to stationary pop
i

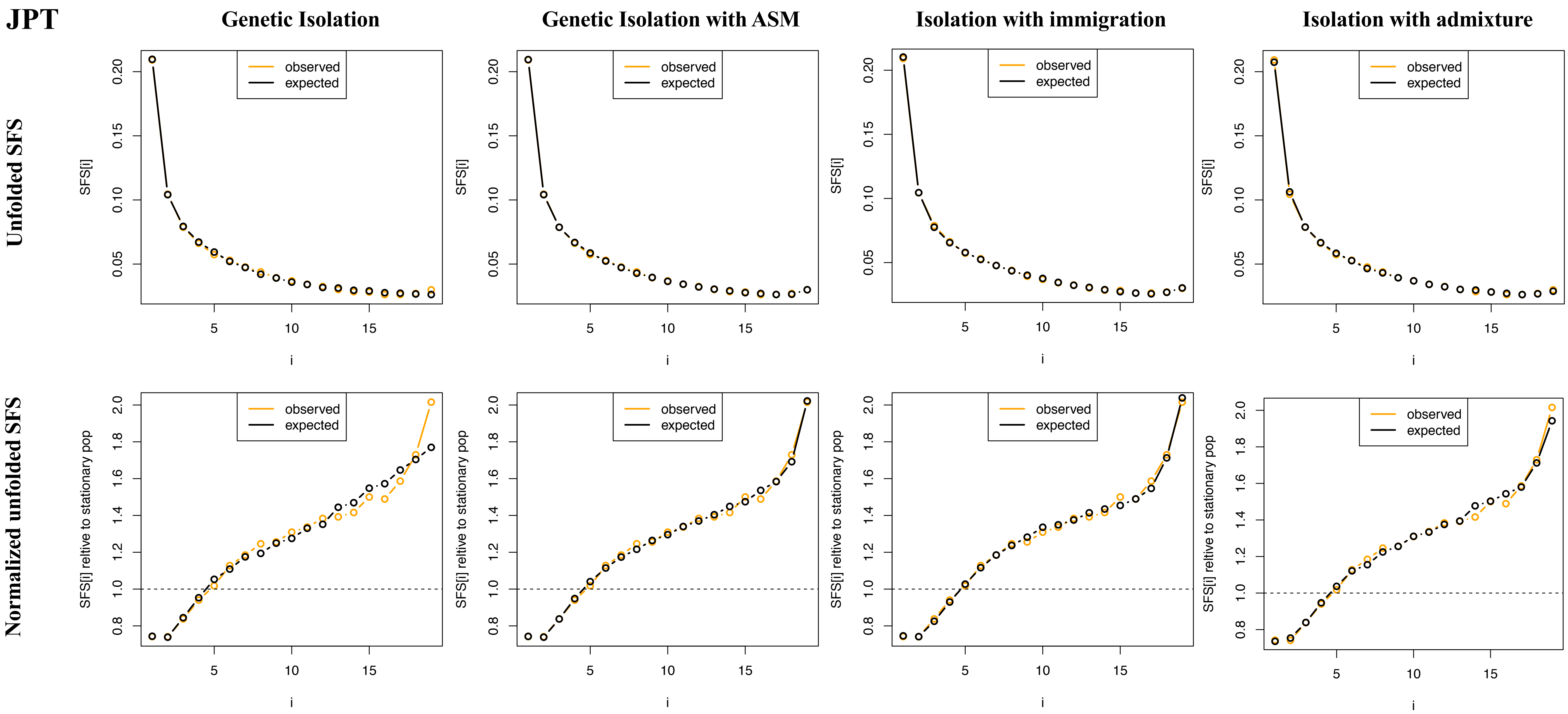
JPT
Genetic Isolation
Genetic Isolation with ASM
Isolation with immigration
Isolation with admixture
Unfolded SFS
Normalized unfolded SFS
observed
expected
SFS[i]
SFS[i] reltive to stationary pop
i

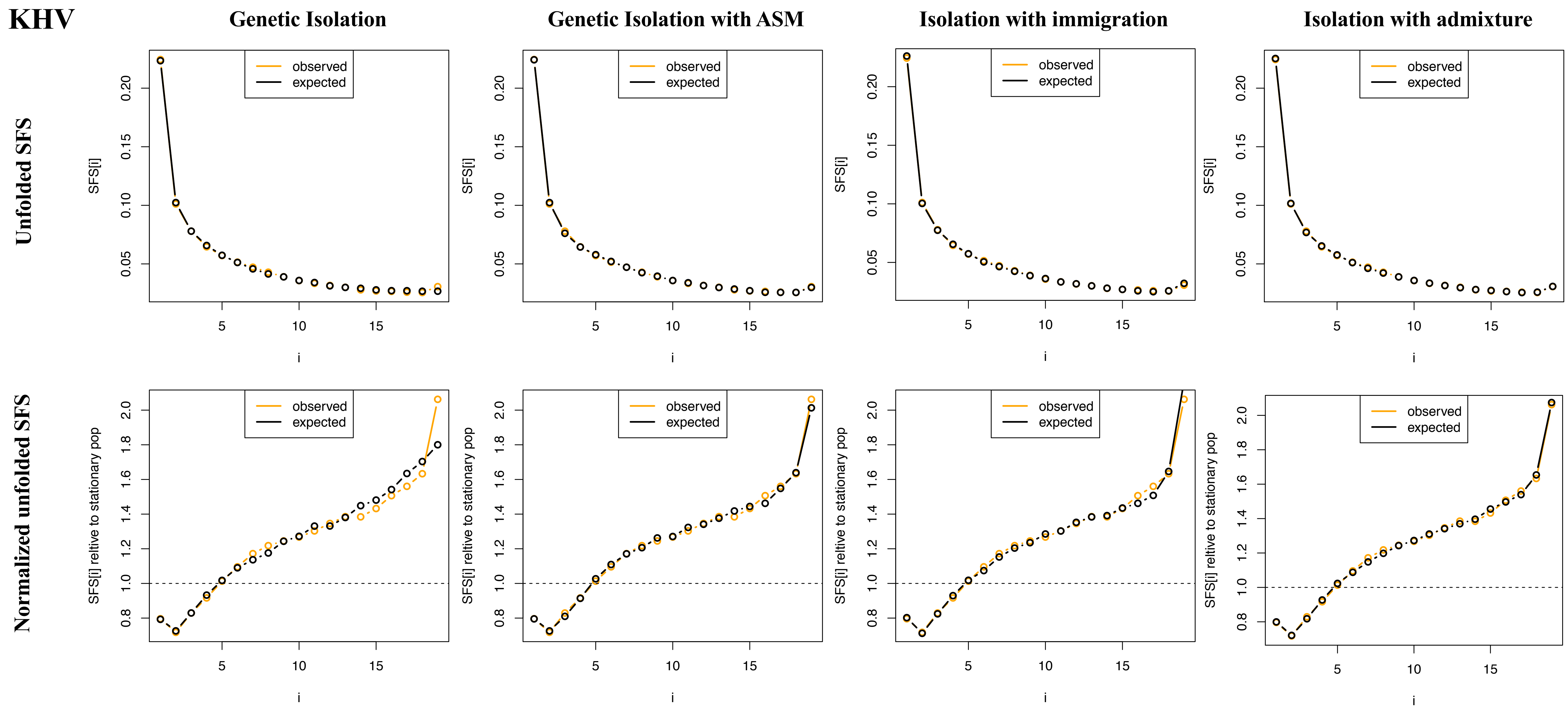
KHV
Genetic Isolation
Genetic Isolation with ASM
Isolation with immigration
Isolation with admixture
Unfolded SFS
Normalized unfolded SFS
SFS[i]
SFS[i] reltive to stationary pop
observed
expected
i
0.05
0.10
0.15
0.20
0.8
1.0
1.2
1.4
1.6
1.8
2.0
5
10
15

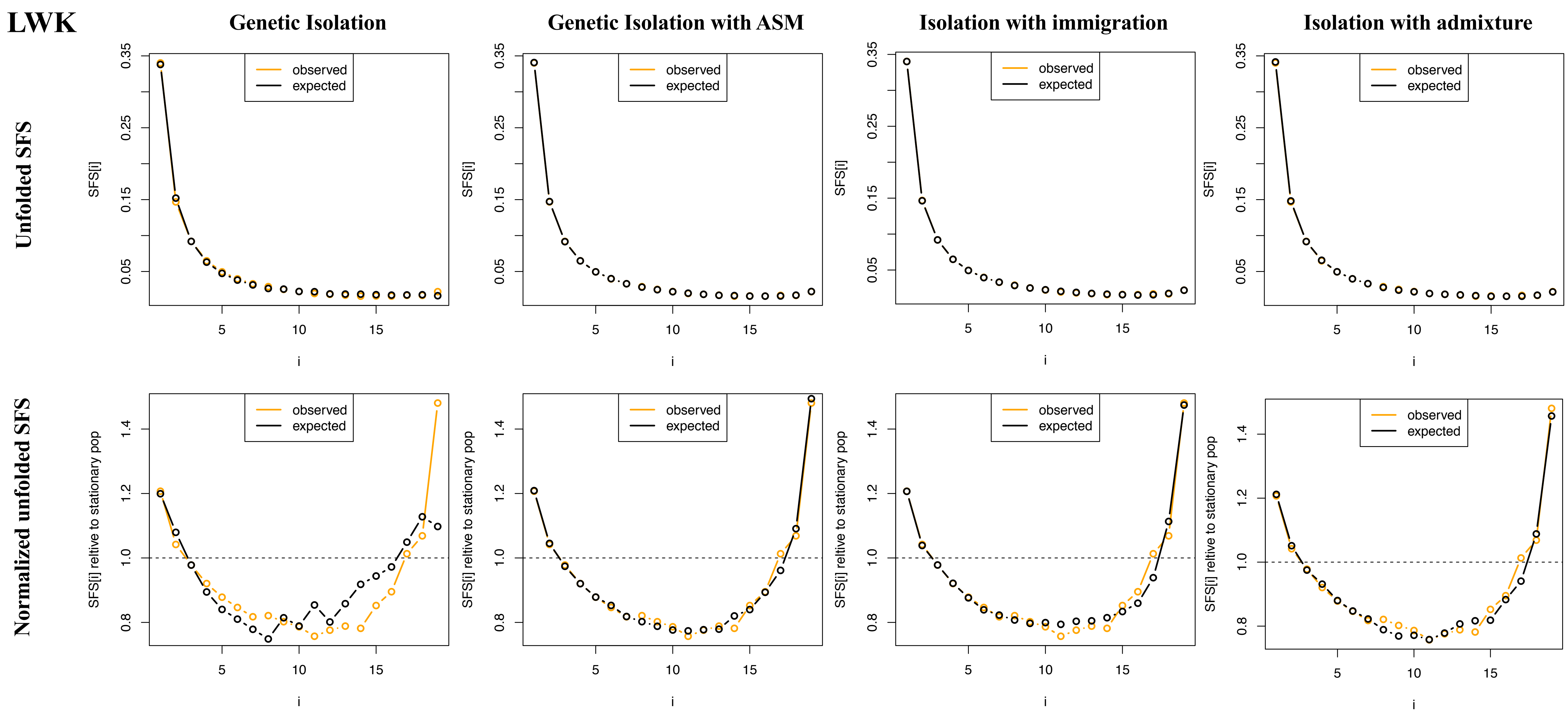

LWK
Genetic Isolation
Genetic Isolation with ASM
Isolation with immigration
Isolation with admixture
Unfolded SFS
Normalized unfolded SFS
observed
expected
SFS[i]
SFS[i] reltive to stationary pop
i

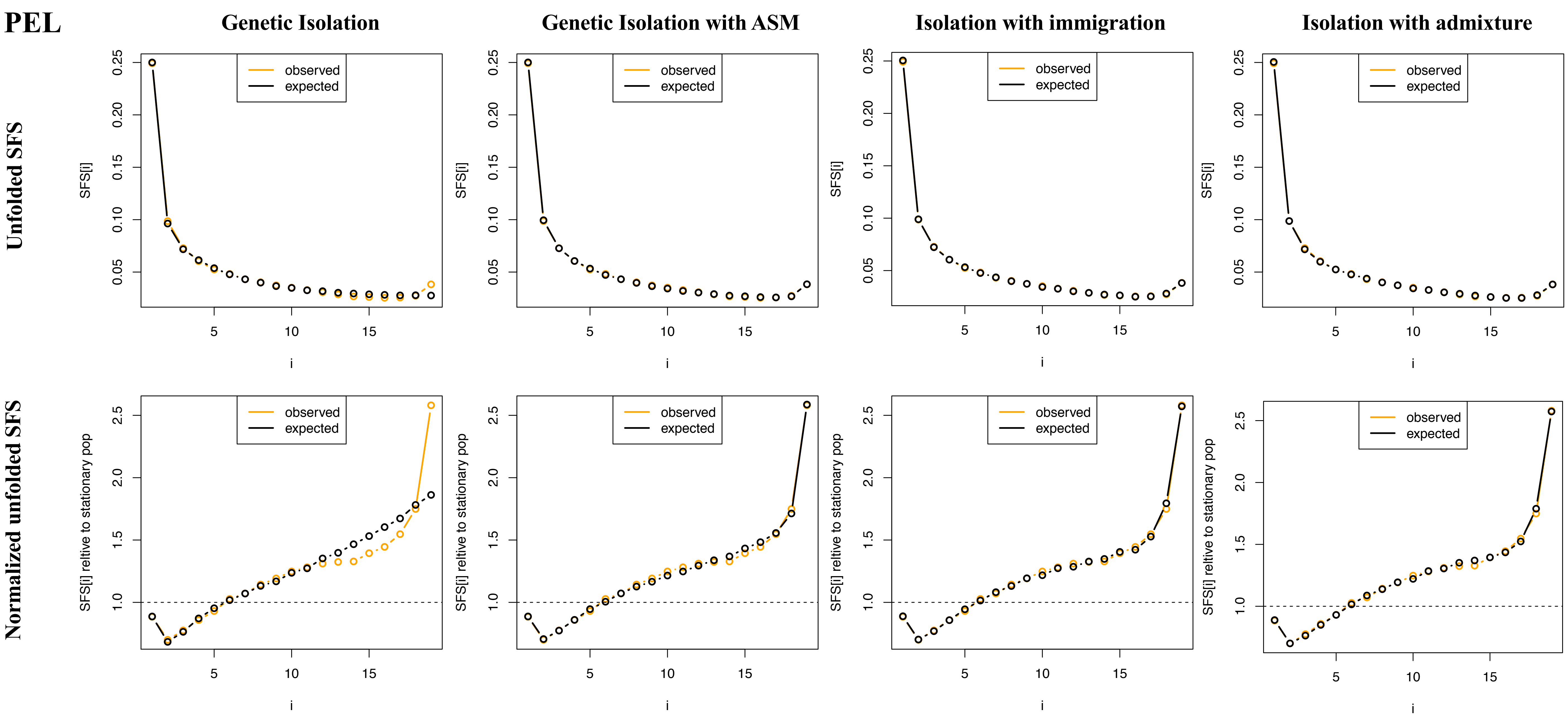
PEL
Genetic Isolation
Genetic Isolation with ASM
Isolation with immigration
Isolation with admixture
Unfolded SFS
Normalized unfolded SFS
observed
expected
SFS[i]
SFS[i] reltive to stationary pop
i

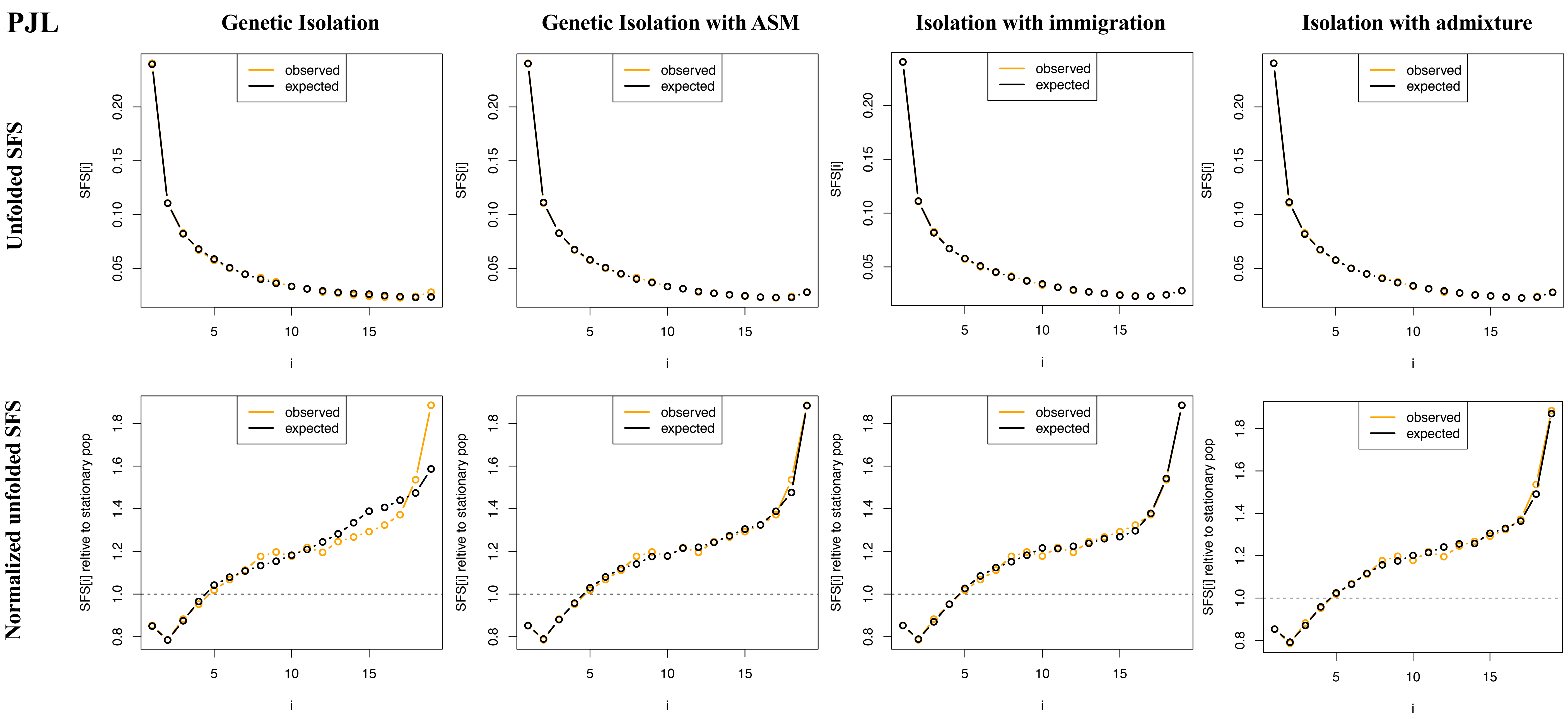
PJL
Genetic Isolation
Genetic Isolation with ASM
Isolation with immigration
Isolation with admixture
Unfolded SFS
Normalized unfolded SFS
observed
expected
SFS[i]
SFS[i] reltive to stationary pop
i

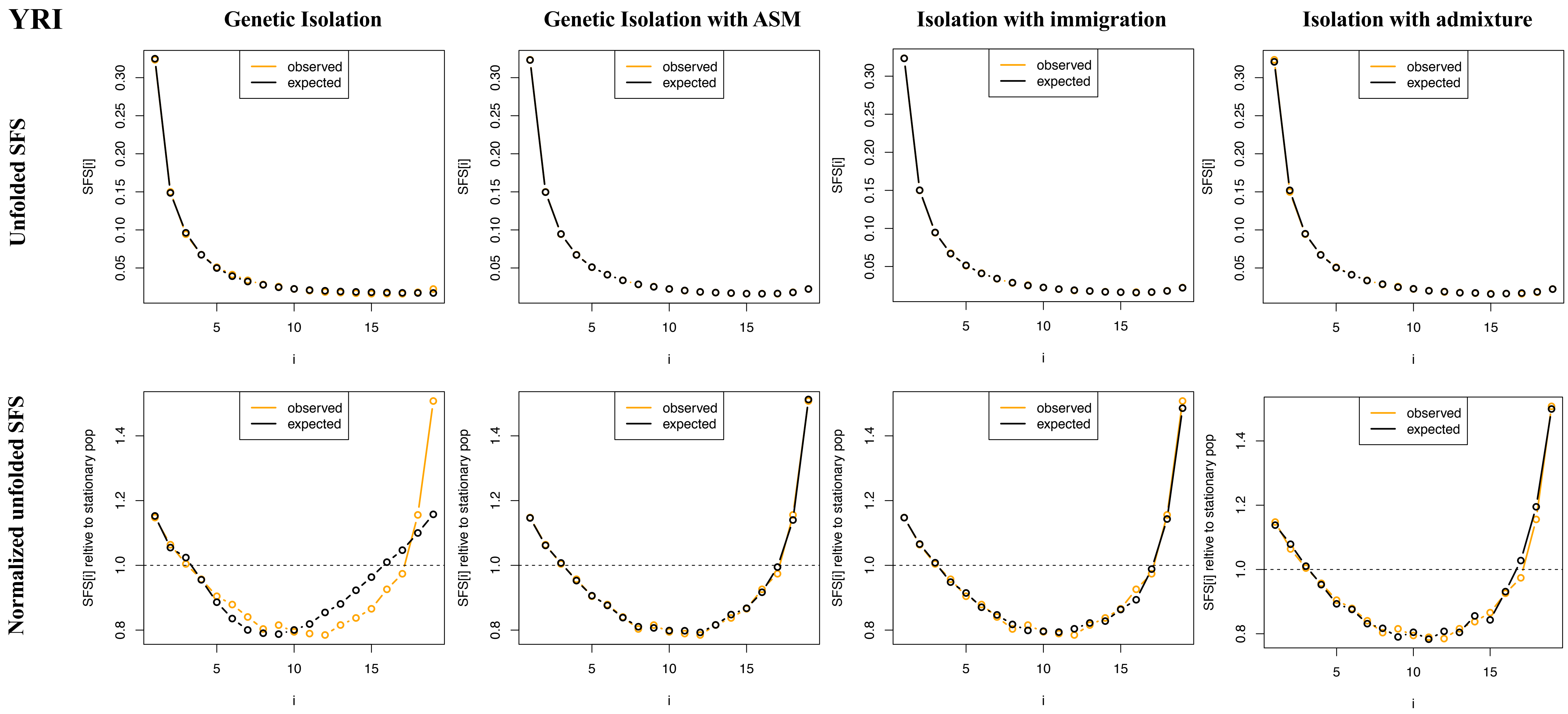
YRI
Genetic Isolation
Genetic Isolation with ASM
Isolation with immigration
Isolation with admixture
Unfolded SFS
Normalized unfolded SFS
observed
expected
SFS[i]
SFS[i] reltive to stationary pop
i
0.05
0.10
0.15
0.20
0.25
0.30
0.8
1.0
1.2
1.4
5
10
15
