# Supplementary material for: Gene flow as a simple cause for an excess of high‐frequency‐derived alleles
Source: Evol Appl. 2020 Jun 2;13(9):2254–63. doi: 10.1111/eva.12998 (PMC7513730; doi:10.1111/eva.12998)

**Supp.** **Information** **12** **–** **Effect of sampling populations exchanging gene flow or not on their joint SFS.** Three populations were simulated under an *IA* model, all with *n* = 10, *N* = 4,000, and $\tau_{DIV}=2.5$. At $\tau_{ADM}=0$, the source population sends genes into the target population at a rate *a*. The “Other” population is a control having no gene flow with the target. Colors are proportional to the SFS entries, in a log scale; in white, empty entries; entry (0;0) has been removed to maximize the differences between the other SFS entries.


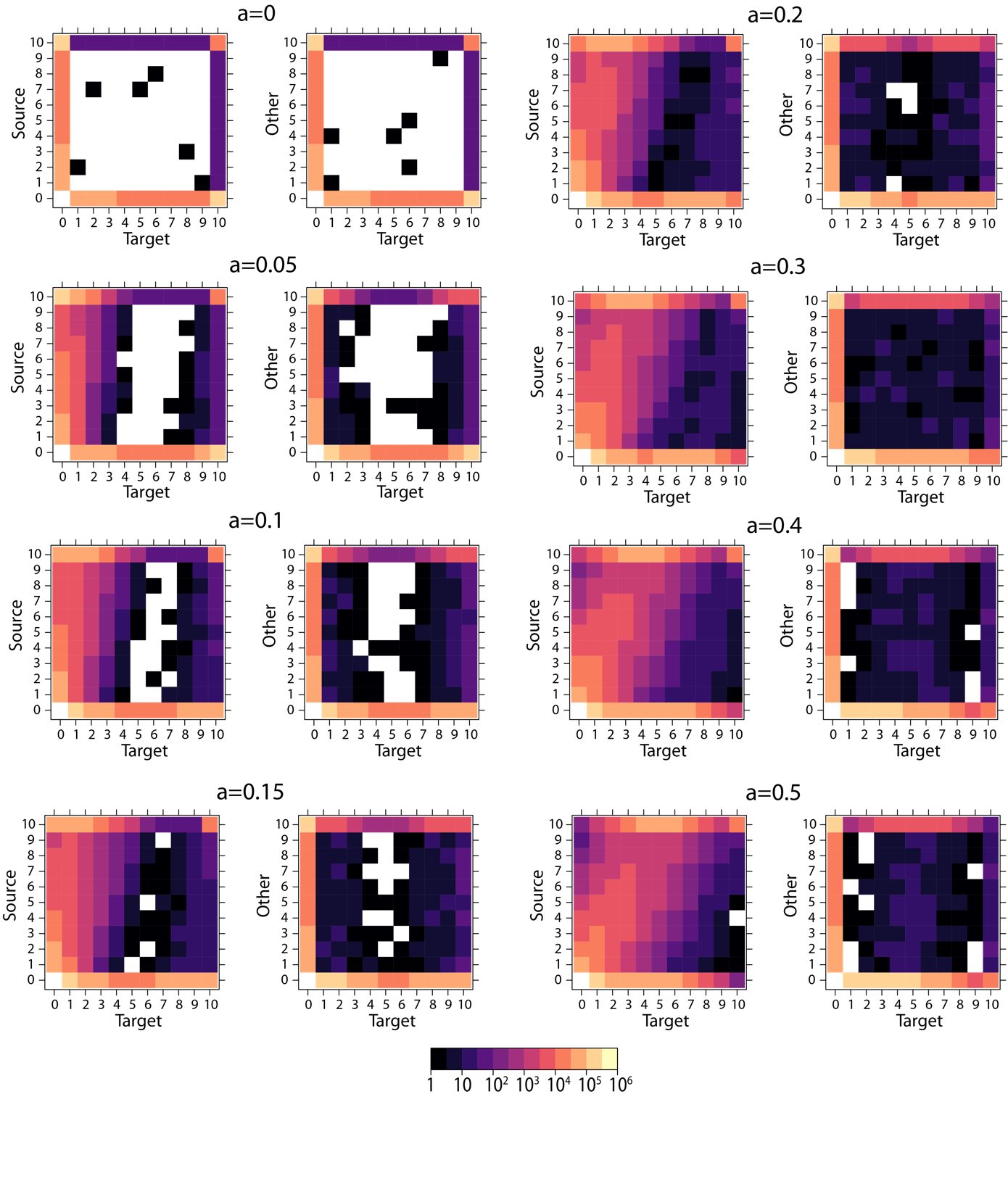

Supplement: Supplementary file 12 — Supplementary Material [file EVA-13-2254-s012.docx]
