# Supplementary material for: Gene flow as a simple cause for an excess of high‐frequency‐derived alleles
Source: Evol Appl. 2020 Jun 2;13(9):2254–63. doi: 10.1111/eva.12998 (PMC7513730; doi:10.1111/eva.12998)

**Supp. Information 13 – Looking for the source of high-frequency variants within the 1000G populations.**

We observe an excess of high-frequency neutral variants in each of the studied 1000G populations, and we have evidenced that they receive some gene flow from some other population. We checked if one of the other nine 1000G populations could be this source population. Based on our simulation results indicating that uSFS are caused by the introgression of ancestral alleles from a source population where they are frequent (mainly SFS entries n=0 and n=1), we calculated the frequency of the sites found at frequency n=19 in the population of interest (labeled in the top-left corner) for each of the nine other populations (A). We find that the sites at high-frequency in each studied population are also at high-frequency in the other 1000Genomes populations and that only a very small fraction (<2%) of the sites are fixed for the ancestral allele or have one copy of the derived allele (n < 2) in some other population.

We also investigated if the uSFS found in each population persists if we exclude the sites found at low frequency (n < 2) in any one of the other 9 populations: B) Neutral SFS per population and C) associated D-tail statistics.

**A)**


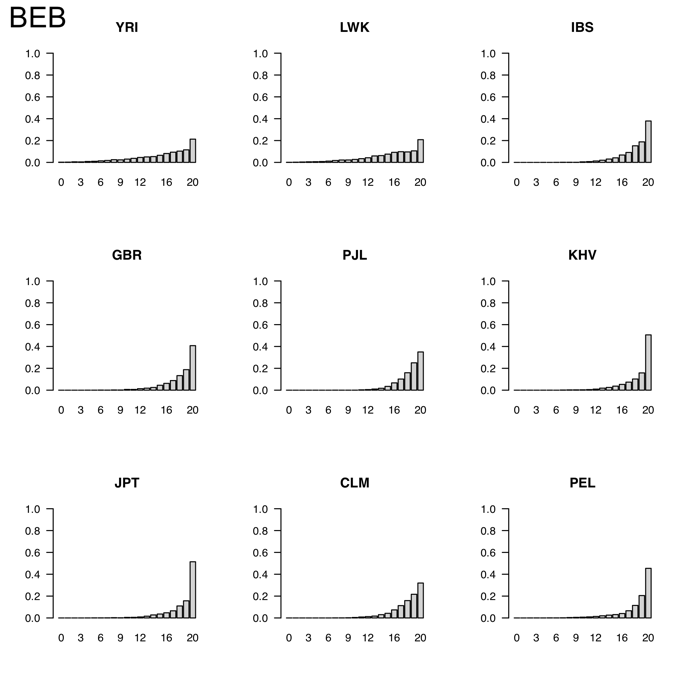

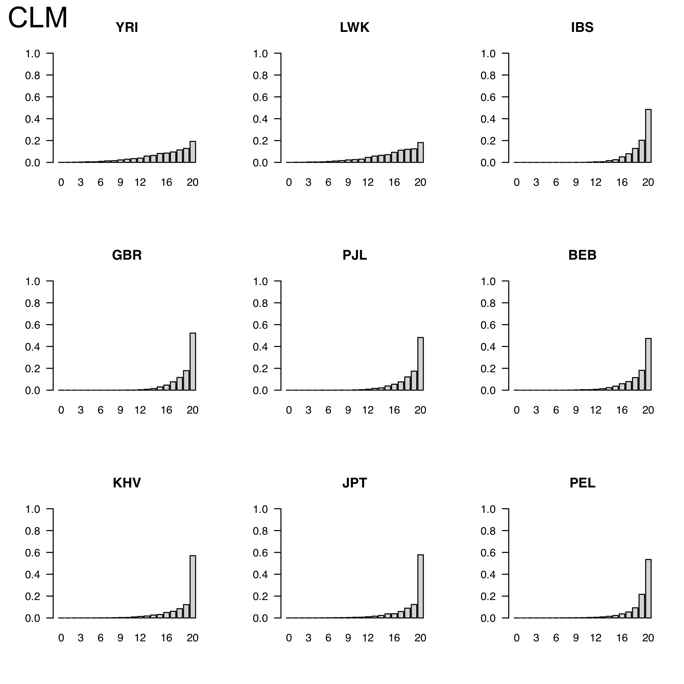

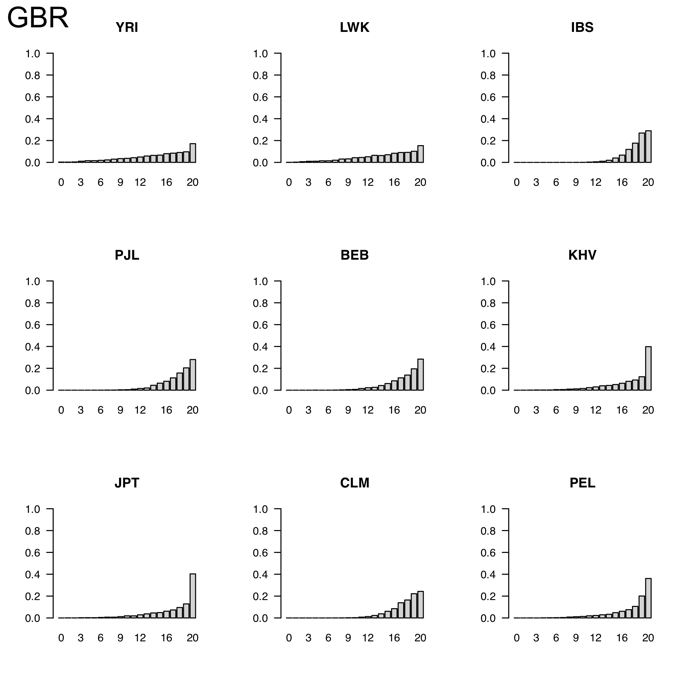

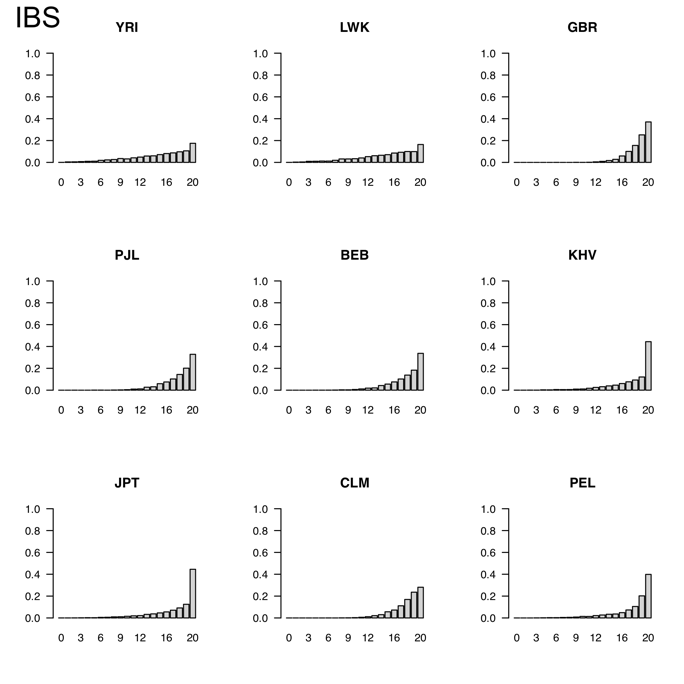

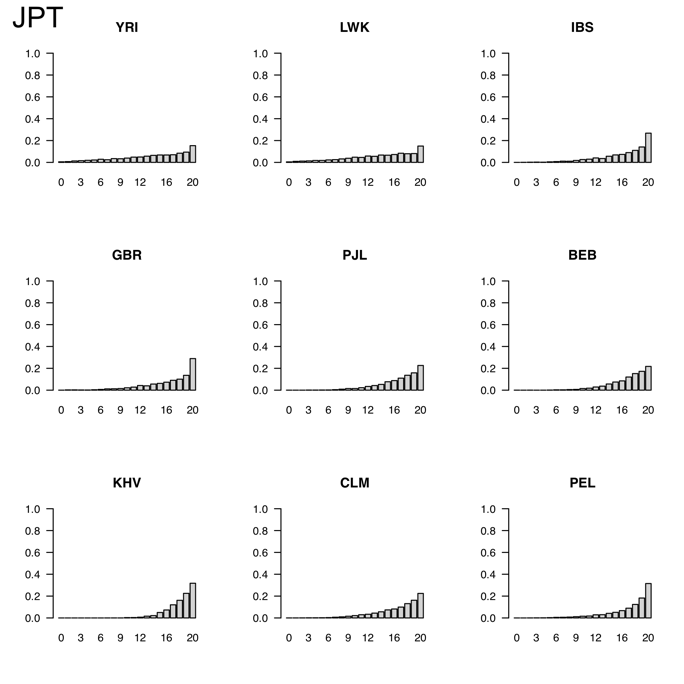

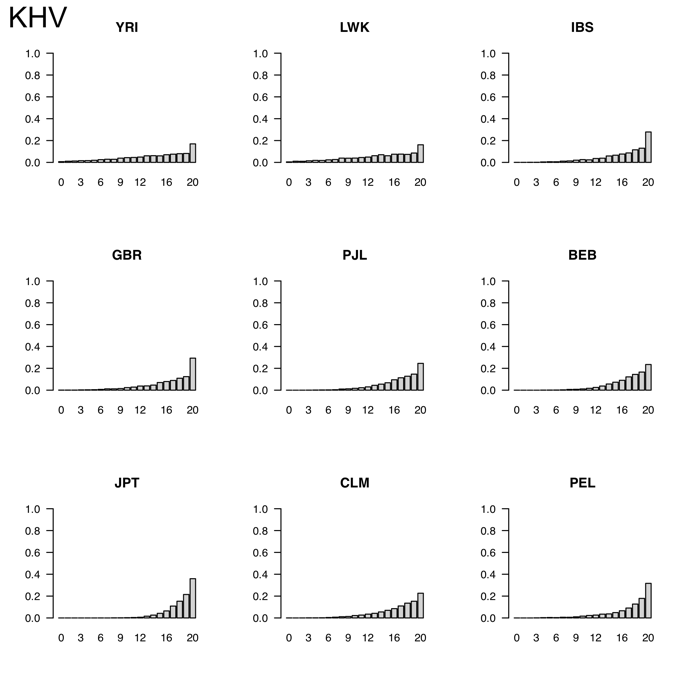

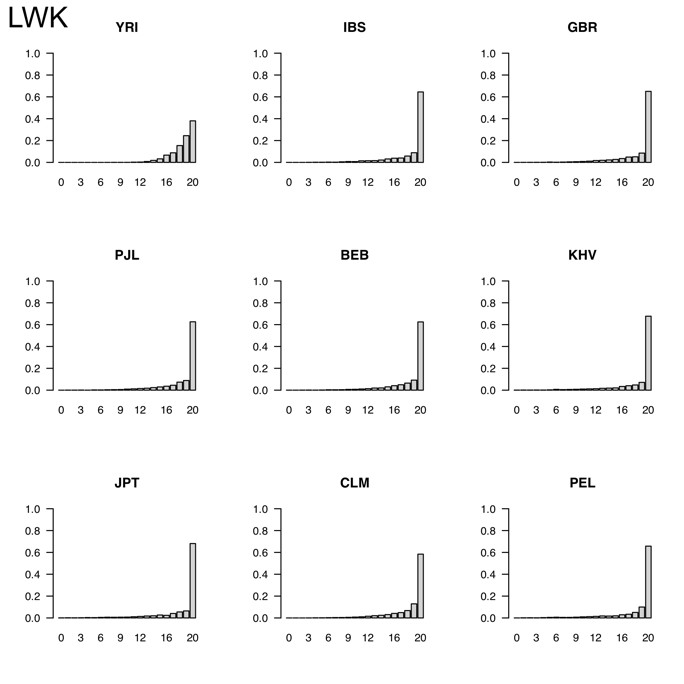

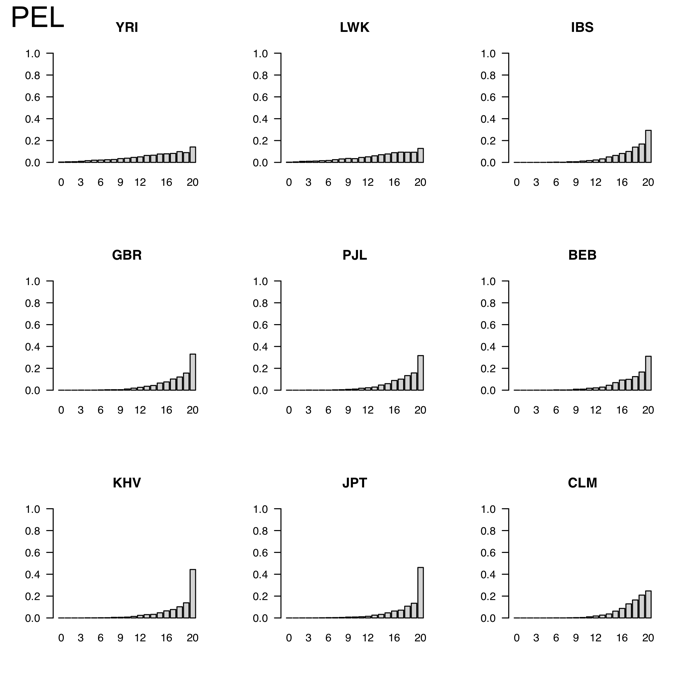

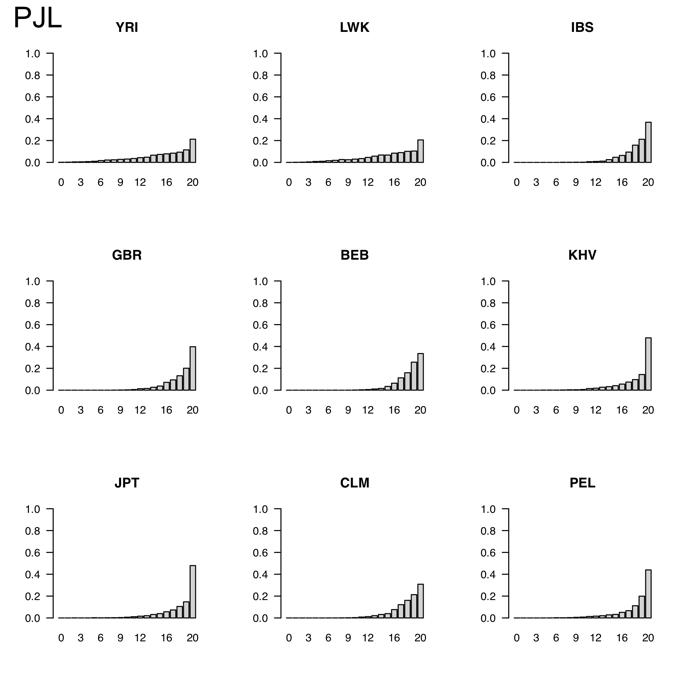

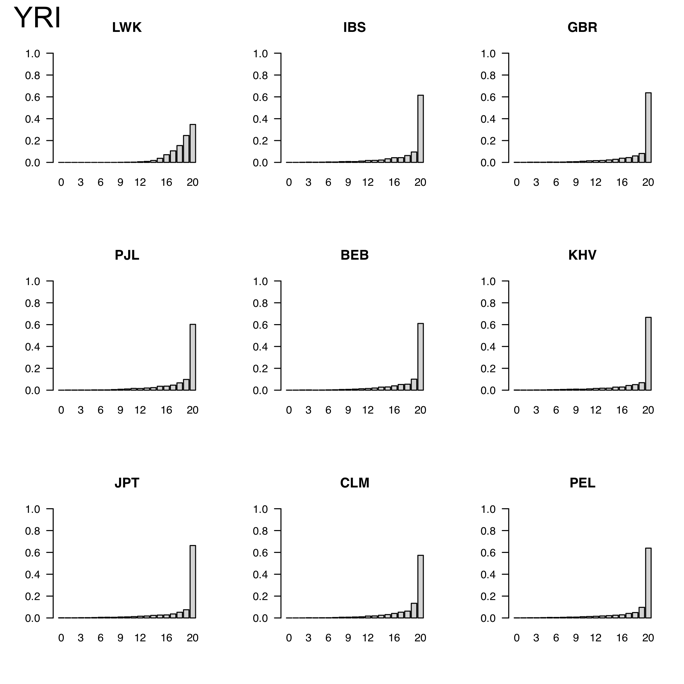


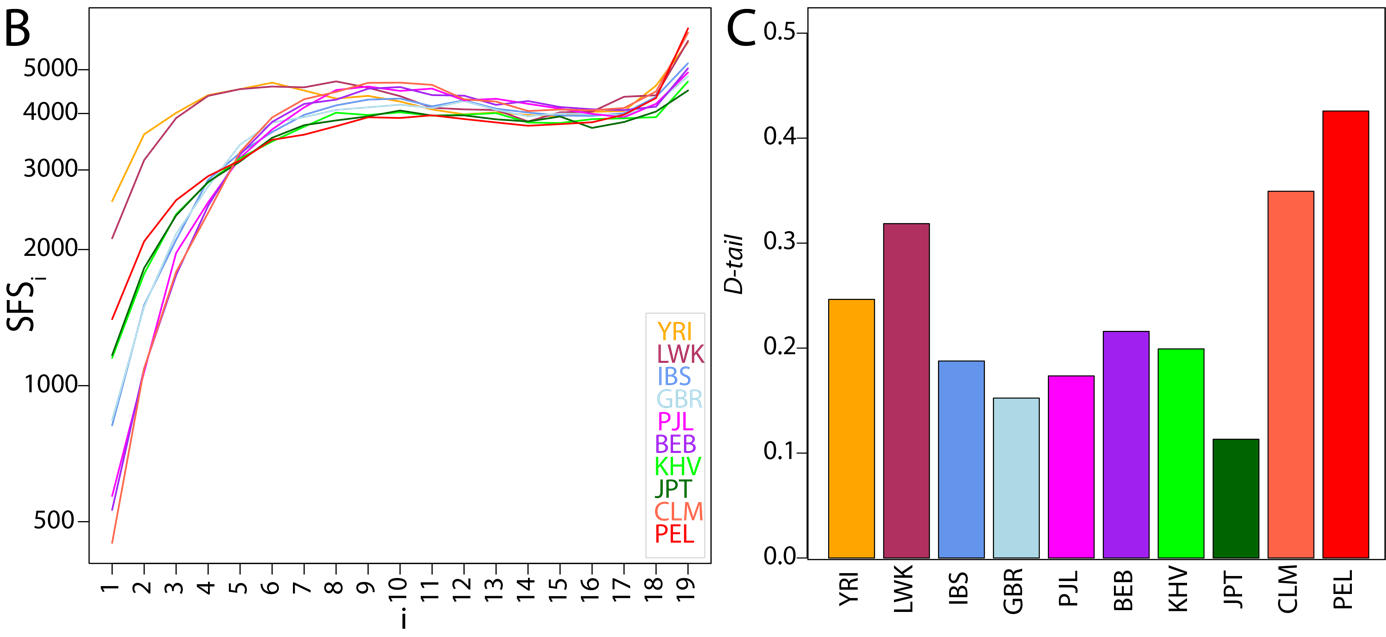

Supplement: Supplementary file 13 — Supplementary Material [file EVA-13-2254-s013.docx]
