# Supplementary material for: Gene flow as a simple cause for an excess of high‐frequency‐derived alleles
Source: Evol Appl. 2020 Jun 2;13(9):2254–63. doi: 10.1111/eva.12998 (PMC7513730; doi:10.1111/eva.12998)

**Supp.** **Information** **14** **–** Effect of range expansion with and without gene flow on SFS properties. SFS was computed from 10 haploid individuals sampled in a population of haploid size 1000, which experienced 50 instantaneous bottlenecks (one every 10 generations). This population is either isolated (in grey) or exchanges continuous gene flow at a rate 0.005 with another population having the same demography and which diverged 510 generations ago (in red). Dots and solid lines were obtained from simulated data sets. Note that we performed boot-straps; however, the 95% block-bootstrap confidence intervals are so small that there are hidden by the solid line and dots.


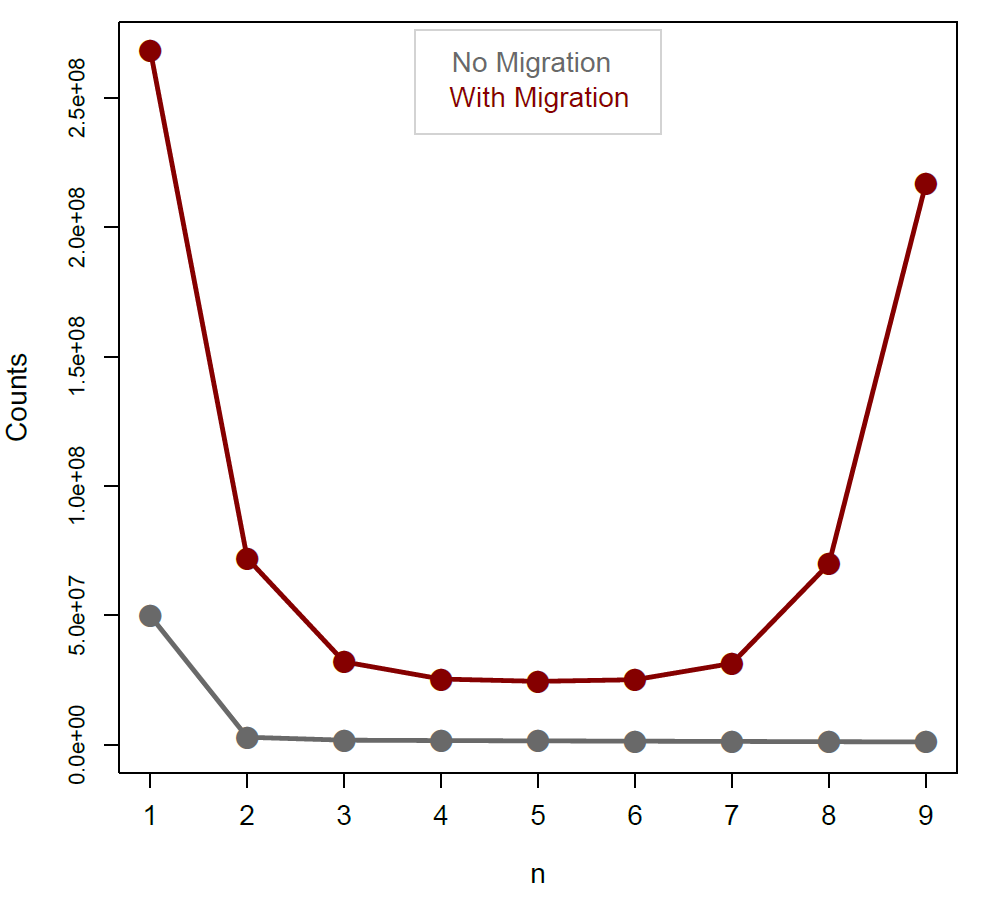

Supplement: Supplementary file 14 — Supplementary Material [file EVA-13-2254-s014.docx]
