# Supplementary material for: Gene flow as a simple cause for an excess of high‐frequency‐derived alleles
Source: Evol Appl. 2020 Jun 2;13(9):2254–63. doi: 10.1111/eva.12998 (PMC7513730; doi:10.1111/eva.12998)
Supplement: Supplementary file 15 — Supplementary Material [file EVA-13-2254-s015.docx]

**Supp.** **Information** **15** **–** Wahlund effect on SFS simulated under an *IA* model for a focal population of haploid sample size *n* = 10 that receives 1/10 migrant from each of *x-1* other source populations (for *x* in [2 : 10]), 0 generations ago. All populations are of sizes *N* = 4,000 and diverged at $\tau_{DIV}=2.5$. $SFS_{i}$is the number of sites with a derived frequency *i*. Dashed lines indicate uSFS; solid lines indicate L or W-shaped SFS.

***
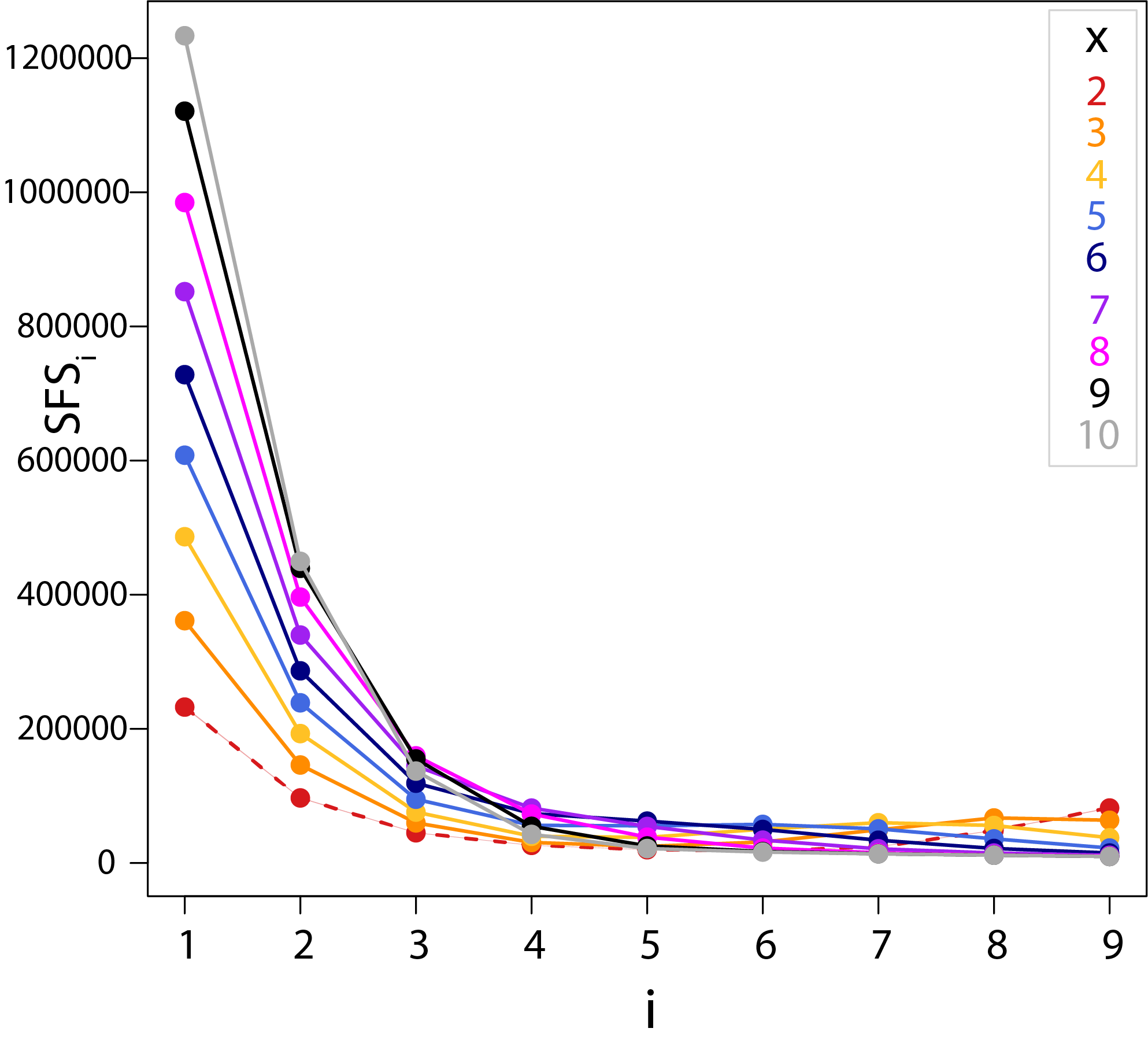
***
